# Supplementary material for: Site-Specific O-Glycosylation Analysis of SARS-CoV-2 Spike Protein Produced in Insect and Human Cells
Source: Viruses. 2021 Mar 25;13(4):551. doi: 10.3390/v13040551 (PMC8064498; doi:10.3390/v13040551)
Supplement: Supplementary file 1 [file viruses-13-00551-s001.zip › Figure S4 Surface exposure analysis.pdf]

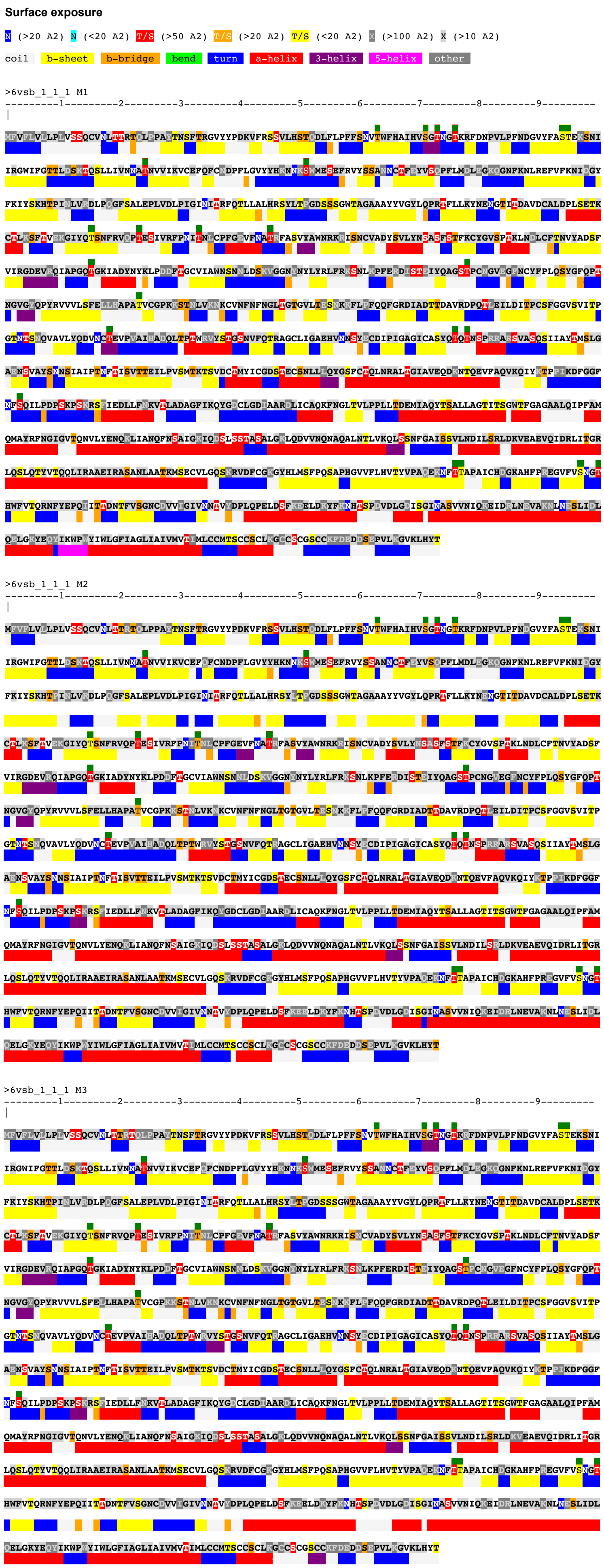

Surface areas of selected residues

Label	A2
A:THR_63	36
B:THR_63	31
C:THR_63	21
A:SER_71	30
B:SER_71	42
C:SER_71	43
A:THR_73	130
B:THR_73	125
C:THR_73	136
A:THR_76	51
B:THR_76	45
C:THR_76	86
A:SER_94	2
B:SER_94	3
C:SER_94	3
A:THR_95	5
B:THR_95	0
C:THR_95	2
A:THR_124	106
B:THR_124	97
C:THR_124	105
A:SER_151	56
B:SER_151	74
C:SER_151	62
A:THR_315	13
B:THR_315	9
C:THR_315	16
A:THR_323	73
B:THR_323	75
C:THR_323	76
A:THR_333	120
B:THR_333	37
C:THR_333	34
A:THR_345	148
B:THR_345	145
C:THR_345	123
A:THR_415	101
B:THR_415	50
C:THR_415	91
A:THR_478	97
B:THR_478	101
C:THR_478	48
A:THR_523	17
B:THR_523	38
C:THR_523	23
A:THR_618	89
B:THR_618	93
C:THR_618	94
A:THR_676	72
B:THR_676	67
C:THR_676	68
A:THR_678	60
B:THR_678	54
C:THR_678	62
A:SER_803	79
B:SER_803	79
C:SER_803	79
A:THR_1076	66
B:THR_1076	65
C:THR_1076	60
A:THR_1077	1
B:THR_1077	1
C:THR_1077	1
A:SER_1097	2
B:SER_1097	2
C:SER_1097	2
A:THR_1100	131
B:THR_1100	129
C:THR_1100	128

Figure S4. Surface exposure analysis. Conformational Analysis Tools (CAT) were used to estimate the solvent accessible surface (SAS) of the amino acids in the 6vsb_1_1_1 model. SAS (upper lines) of the three individual subunits of protein S are shown in the context of secondary structure (lower lines). Color code for SAS range in square angstroms and secondary structure are indicated. Small dark green squares above the sequence indicate O-glycosylated amino acids identified in the study. SAS values for these select positions are shown in the table on the right.
