# Supplementary material for: Site-Specific O-Glycosylation Analysis of SARS-CoV-2 Spike Protein Produced in Insect and Human Cells
Source: Viruses. 2021 Mar 25;13(4):551. doi: 10.3390/v13040551 (PMC8064498; doi:10.3390/v13040551)
Supplement: Supplementary file 1 [file viruses-13-00551-s001.zip › Captions for supplementary datasets.pdf]

Captions for supplementary datasets

Dataset S1. List of peptides identified with Orbitrap Fusion Lumos mass spectrometer for the three different S proteins. Tabs with peptide groups, as well as PSMs are included for each different protein and digestion approach (i1 – monomeric RBD expressed in insect cells, i3 – ectodomain expressed in insect cells, h3m – ectodomain expressed in HEK 293F cells, Chy – chymotrypsin, GluTry – Glu-C followed by trypsin). Identified O-glycosites were validated by manual inspection of relevant spectra, and positions indicated at "Manual validation of PTM assignment" columns.

Dataset S2. List of peptides identified with Q Exactive HF-X mass spectrometer for S protein expressed in HEK 293F cells.

Dataset S3. Quantification of O-glycosite occupancy for Orbitrap Fusion Lumos derived data for insect RBD.

Dataset S4. Quantification of O-glycosite occupancy for Orbitrap Fusion Lumos derived data for insect ectodomain.

Dataset S5. Quantification of O-glycosite occupancy for Orbitrap Fusion Lumos derived data for HEK 293F ectodomain.

Dataset S6. Quantification of O-glycosite occupancy for Q Exactive HF-X derived data.

Dataset S7. Examples of HCD MS2 spectra representing the different (glyco)peptides identified using the Q Exactive HF-X mass spectrometer. The zipped file contains spectra of O-glycosylated peptides included in occupancy quantification (Figure 3b and Dataset S6) of data derived from the Q Exactive HF-X experiments. Examples of non-O-glycosylated peptide spectra are also included. The naming of the files follows the following format O-glycosite position-number-Y/N, where the number indicates the order number in Dataset S6, and the Y/N informs, whether the peptide is O-glycosylated (Y - yes), or not (N - no).
