# Supplementary material for: Site-Specific O-Glycosylation Analysis of SARS-CoV-2 Spike Protein Produced in Insect and Human Cells
Source: Viruses. 2021 Mar 25;13(4):551. doi: 10.3390/v13040551 (PMC8064498; doi:10.3390/v13040551)

Figure S3. Examples of ETD MS2 spectra representing the different O-glycosites identified using the Orbitrap Fusion Lumos mass spectrometer. c and z product fragment ions are annotated in the peptide sequence. The source raw file is indicated in the top left corner (i1 – monomeric RBD expressed in insect cells, i3 – ectodomain expressed in insect cells, h3m – ectodomain expressed in HEK 293F cells).

T63

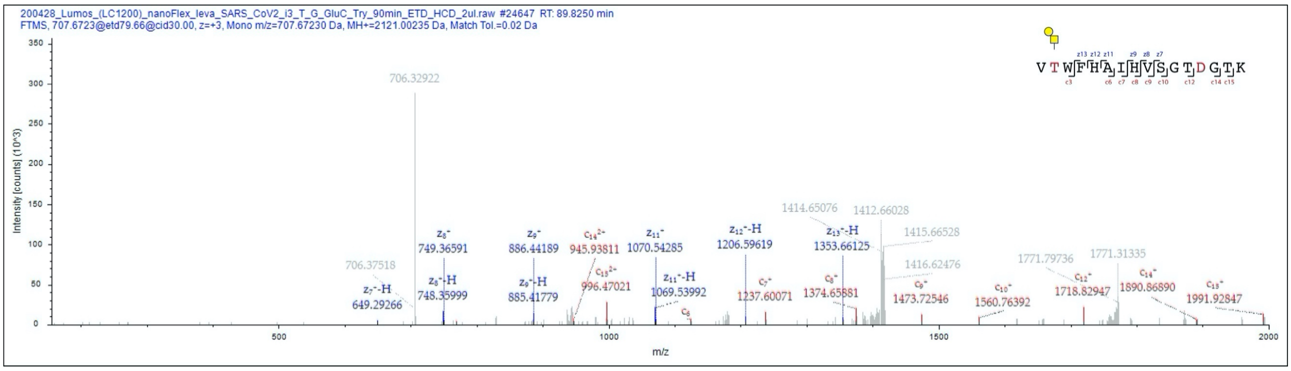

S71, T73

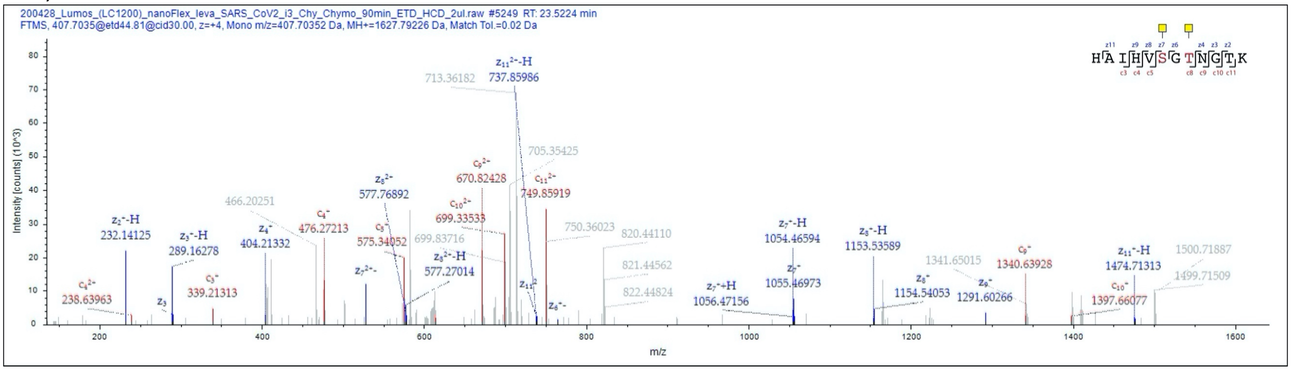

T73, T76

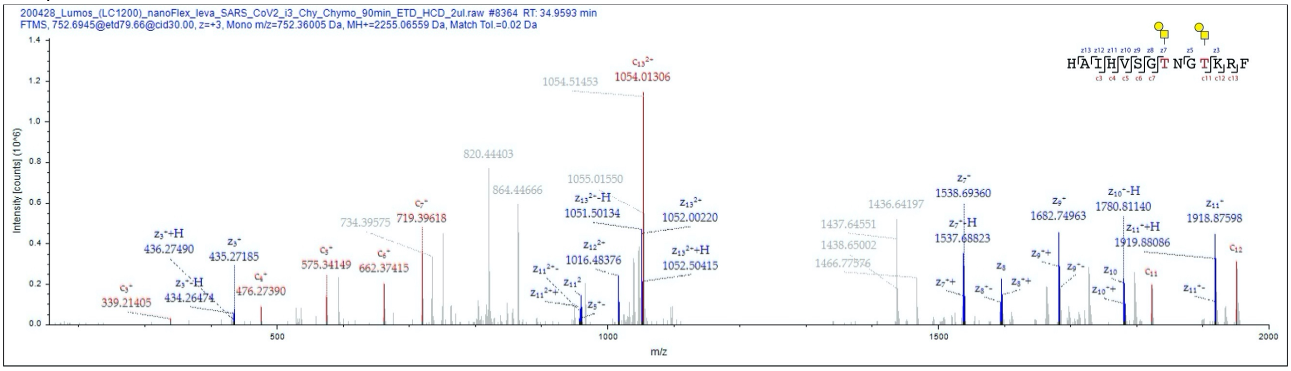

94-95 (1x)

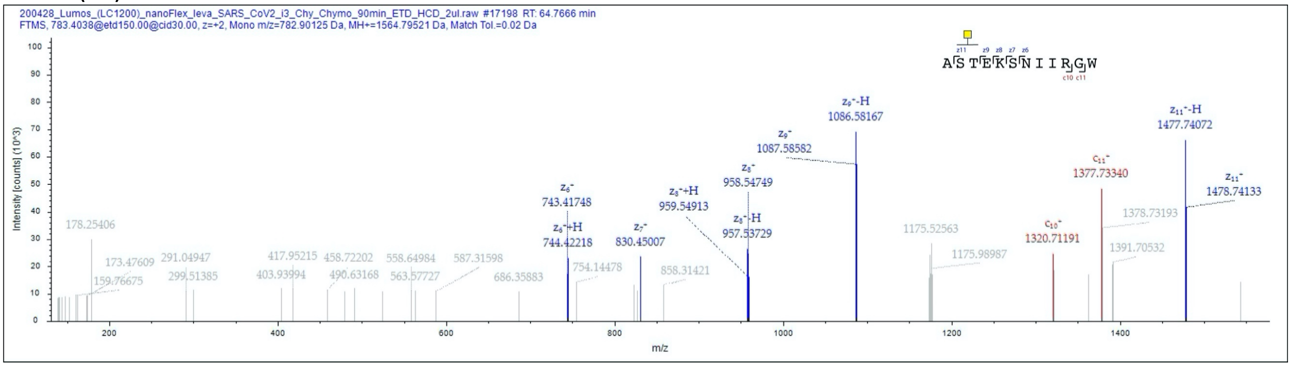

T124

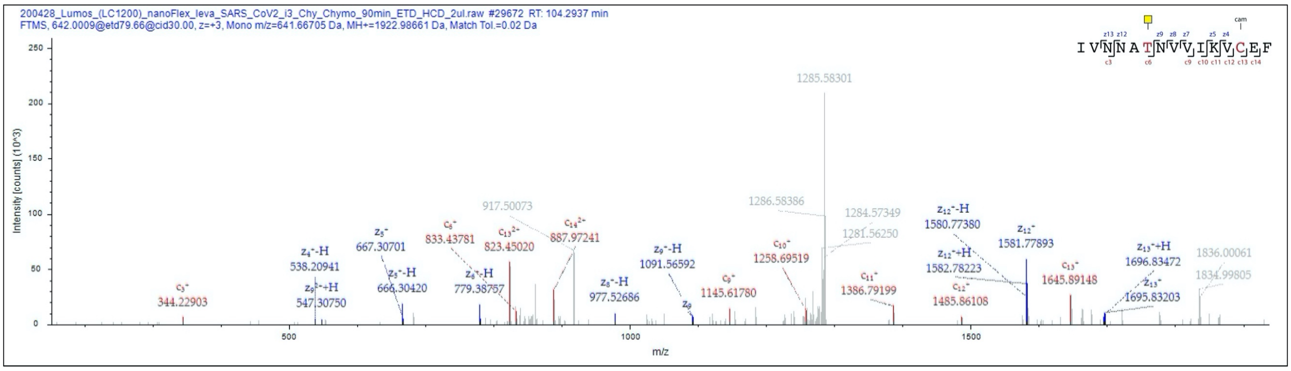

S151

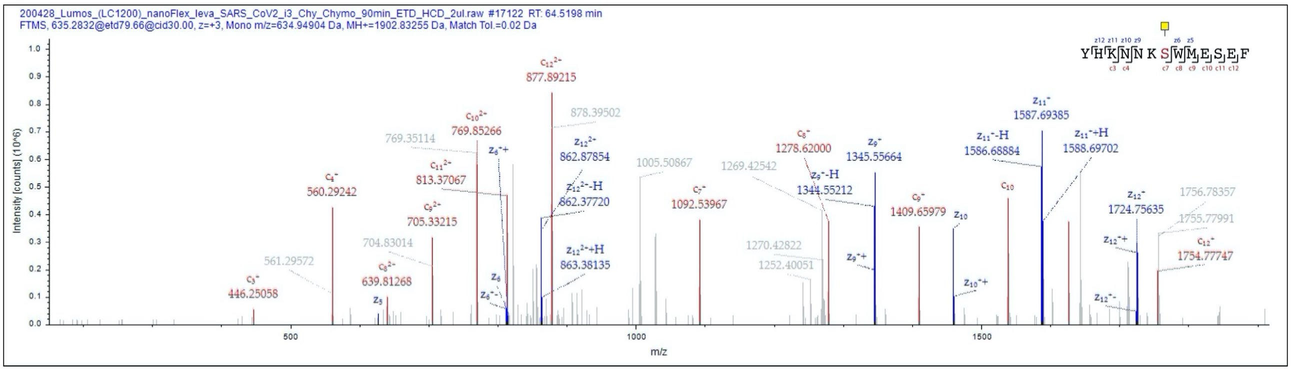

T315

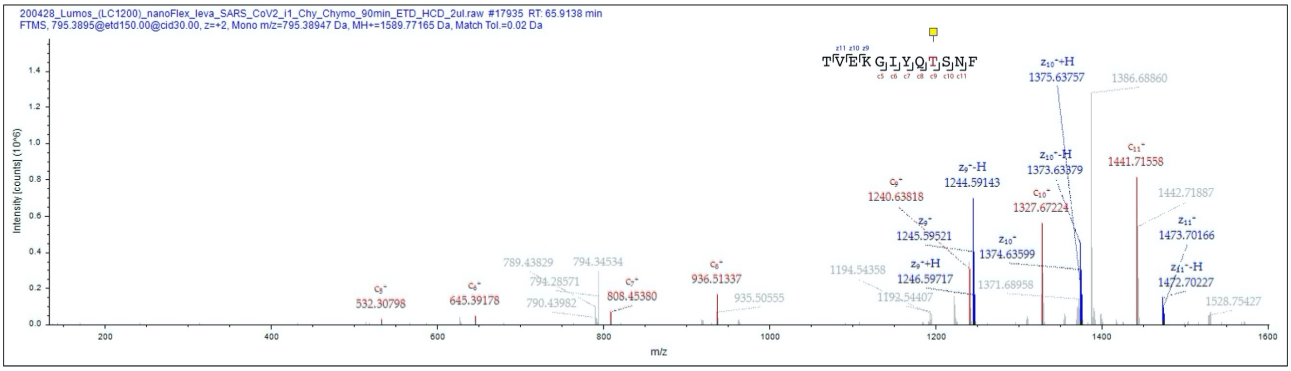

T323

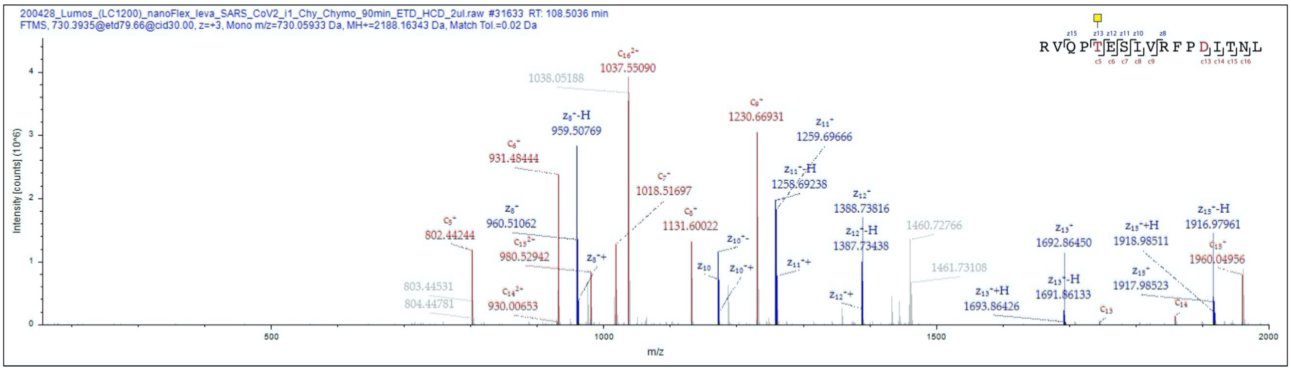

T333

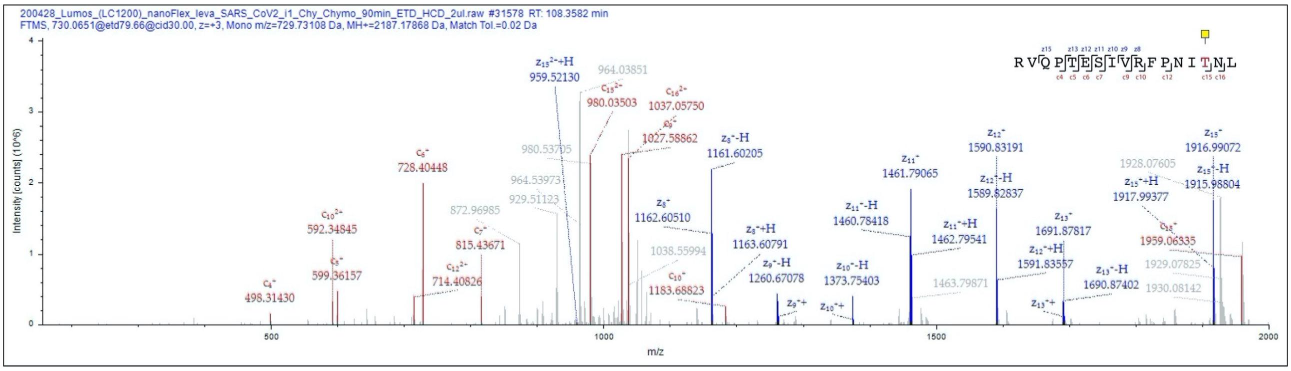

T345

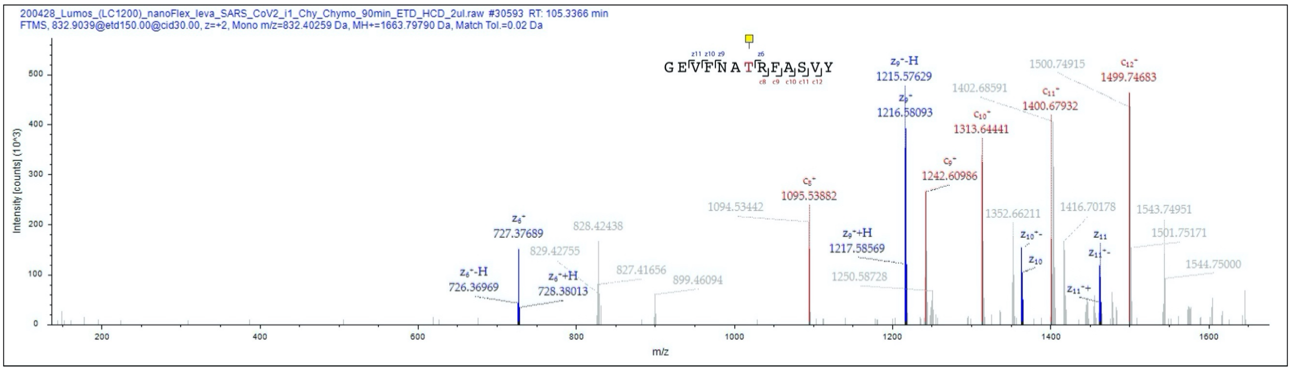

T415

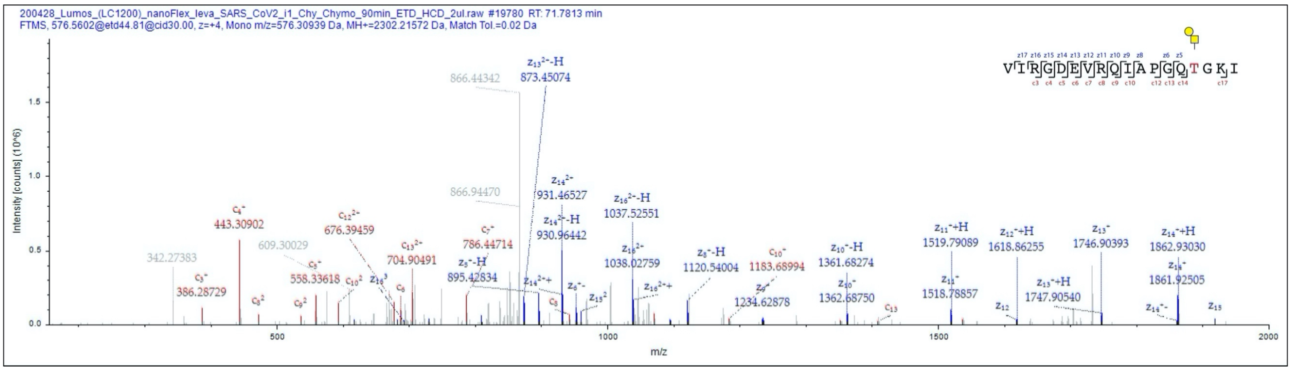

T478

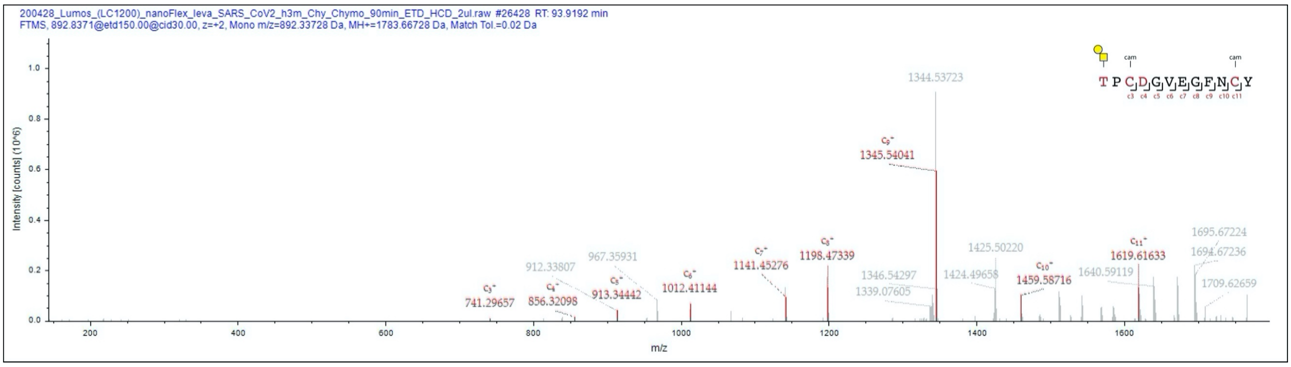

T523

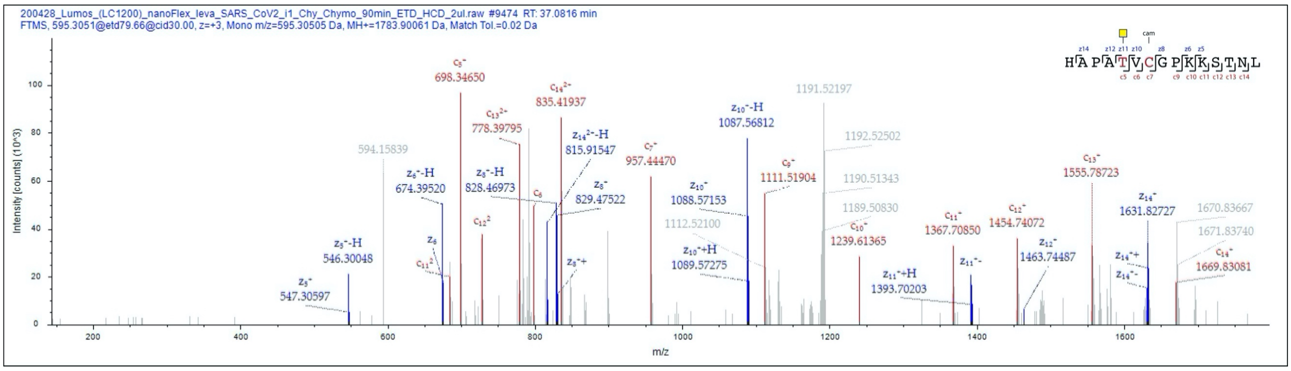

T618

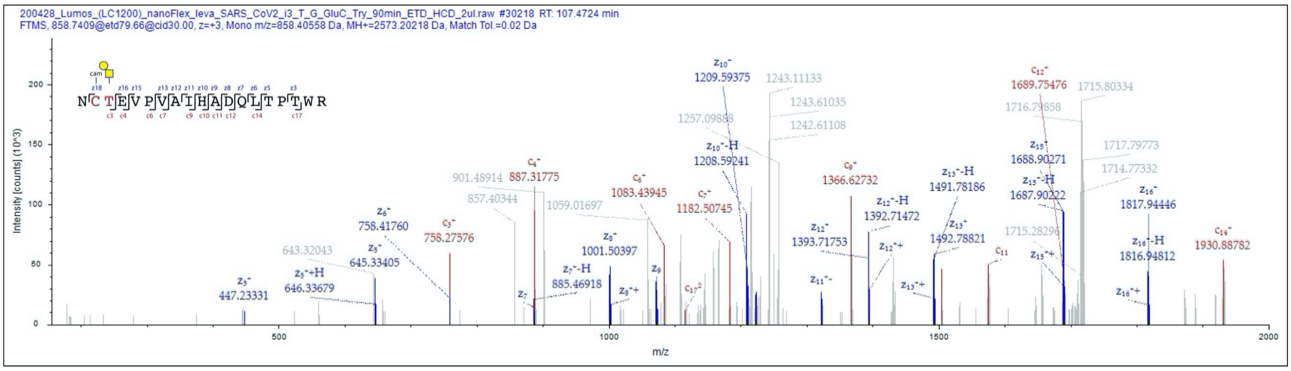

T676, T678

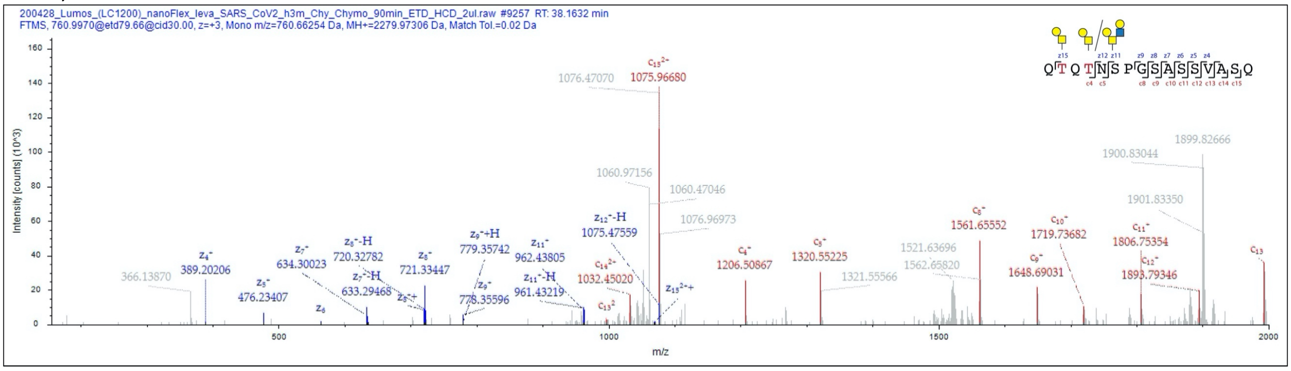

S803

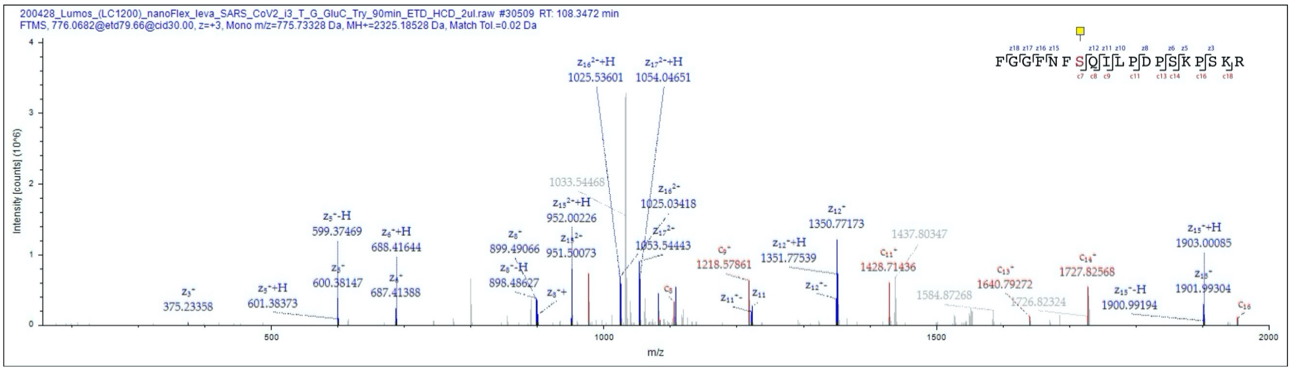

T1076, T1077

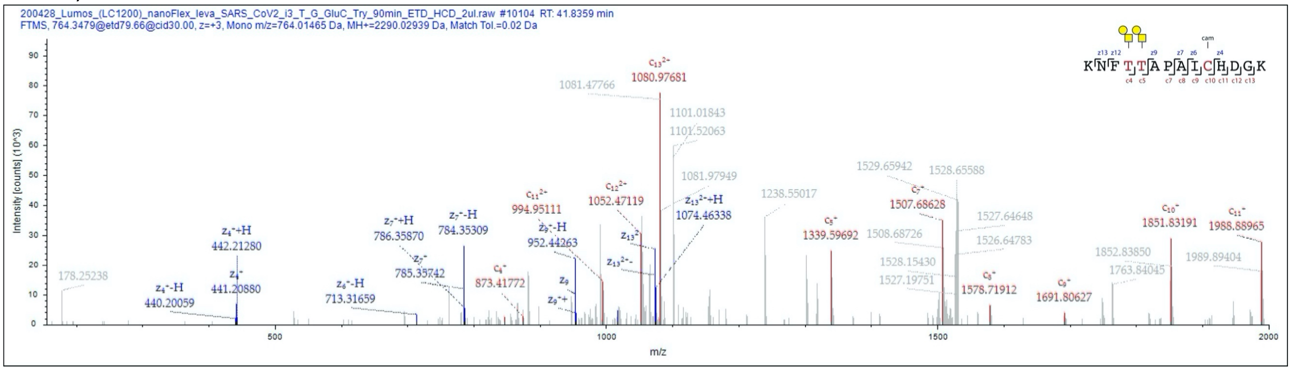

T1097

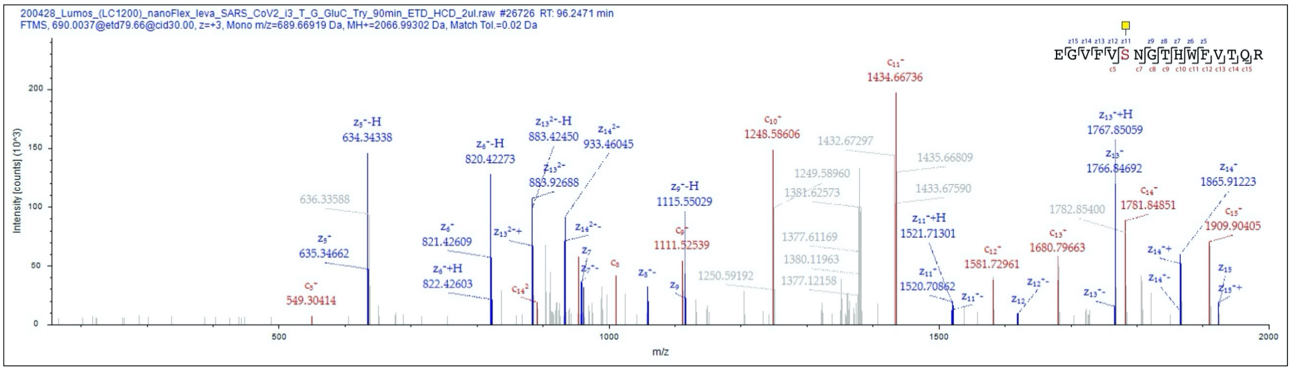

T1097, T1100

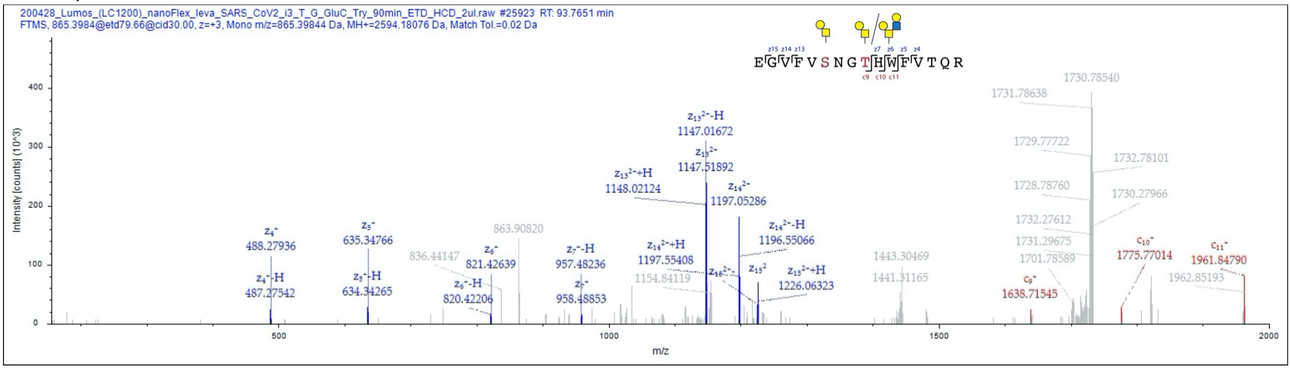

Supplement: Supplementary file 1 [file viruses-13-00551-s001.zip › Figure S3 Spectra Orbitrap Fusion Lumos ETD.pdf]
