# Supplementary material for: Site-Specific O-Glycosylation Analysis of SARS-CoV-2 Spike Protein Produced in Insect and Human Cells
Source: Viruses. 2021 Mar 25;13(4):551. doi: 10.3390/v13040551 (PMC8064498; doi:10.3390/v13040551)
Supplement: Supplementary file 1 [file viruses-13-00551-s001.zip › Figures S1 and S2 In gel digestion and total ion chromatograms.pdf]

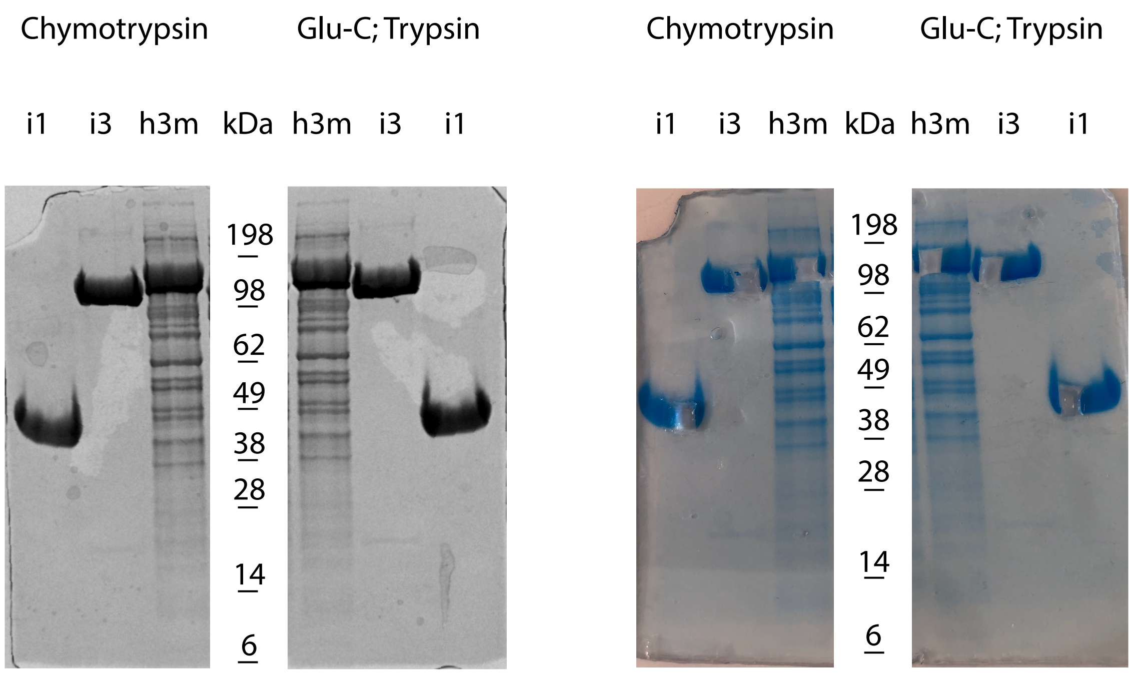

Figure S1. In gel digestion of S proteins. 20 µg per lane of of SARS- CoV-2 S monomeric RBD expressed in insect cells (i1), ectodomain expressed in insect cells (i3), and ectodomain expressed in HEK 293F cells (h3m) were separated on a 4-12 % Bis-Tris gradient gel in 1x MES buffer at 150 V for 1 hour on ice followed by 30 min at 200 V in duplicates and stained with InstantBlue® protein stain. One set of proteins was used for chymotrypsin digestion, whereas the other was used for Glu-C followed by trypsin digestion. The image on the right shows excised gel regions for downstream analysis.

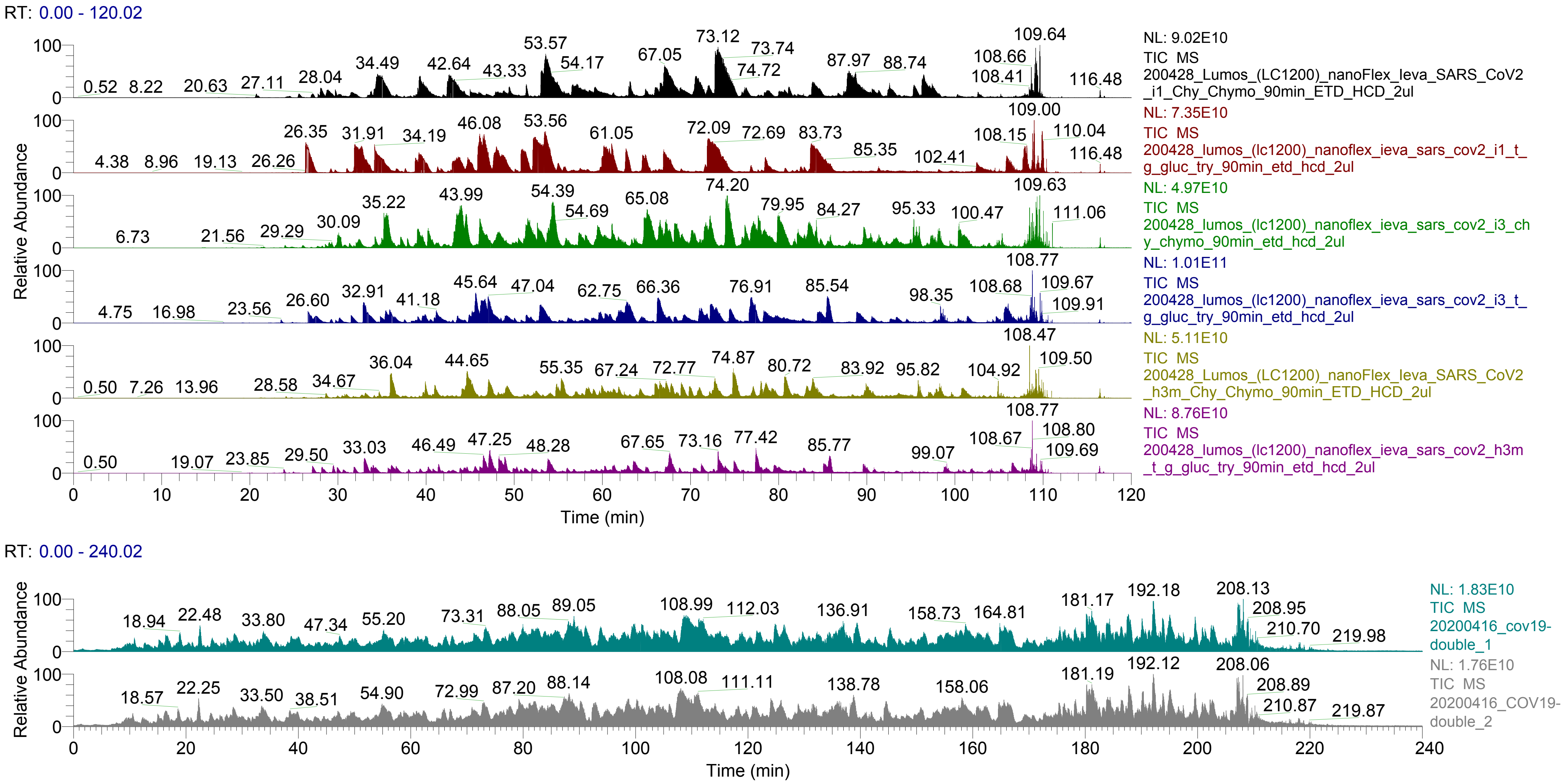

Figure S2. Total ion chromatograms of analyzed samples. Raw files were opened using Thermo Xcalibur Browser and total ion chromatograms (TIC) exported. (a) 2 out of 12 µL of each desalted sample was injected to a Thermo Fisher nLC-MS/MS Fusion Lumos Trybrid mass spectrometer. The peptides were eluted over a 120 min gradient. TIC of the six different samples (Fig. S1) over time are shown. (b) TIC of HEK 293F expressed S samples digested in solution analyzed on a Thermo Fisher Q Exactive HF-X mass spectrometer in duplicate. The peptides were eluted over a 240 min gradient.
