# Supplementary material for: Site-Specific O-Glycosylation Analysis of SARS-CoV-2 Spike Protein Produced in Insect and Human Cells
Source: Viruses. 2021 Mar 25;13(4):551. doi: 10.3390/v13040551 (PMC8064498; doi:10.3390/v13040551)
Supplement: Supplementary file 1 [file viruses-13-00551-s001.zip › Dataset S7. Spectra Q Exactive.zip!/spectrum list-Exp_2/T76-2-Y.pdf]

Fragment Ion View

Project name: 20200330COV19
Sample name: 20200416_COV19-double_2
Created date: 2020-04-18 15:22:56.0
Search name:
Activation type: CID

Peptide sequence: F.HAIHVSGTNGT(203.079373)K.R
Scan number: 6950
Calculated M+H: 1424.7129
Measured M+H: 1424.7179
Charge state: 2
File name: 20200416_COV19-double_2
search type: light

Ions:
a 1+ 2+ 3+
b 1+ 2+ 3+
c 1+ 2+ 3+
x 1+ 2+ 3+
y 1+ 2+ 3+
z 1+ 2+ 3+
[Deselect All]

Neutral Loss:
H_2O (o)
NH_3 (*)
H_3PO_4 (^)
Precursor (#)
Precursor with reduced charges

Mass Type: Mono Avg

Mass Tol: 20
Dalton PPM

Update

Peak Assignment:
Most Intense
Nearest Match
Peak Detect
Precursor Removal

Peak Labels:
Ion m/z
None

Width: 750

Height: 450

HAIHVSGTNGTK, MH+ 1424.7128, m/z 712.8601
File: 20200416_COV19-double_2, Scan: 6950, Precursor m/z: 712.8626, Charge: 2

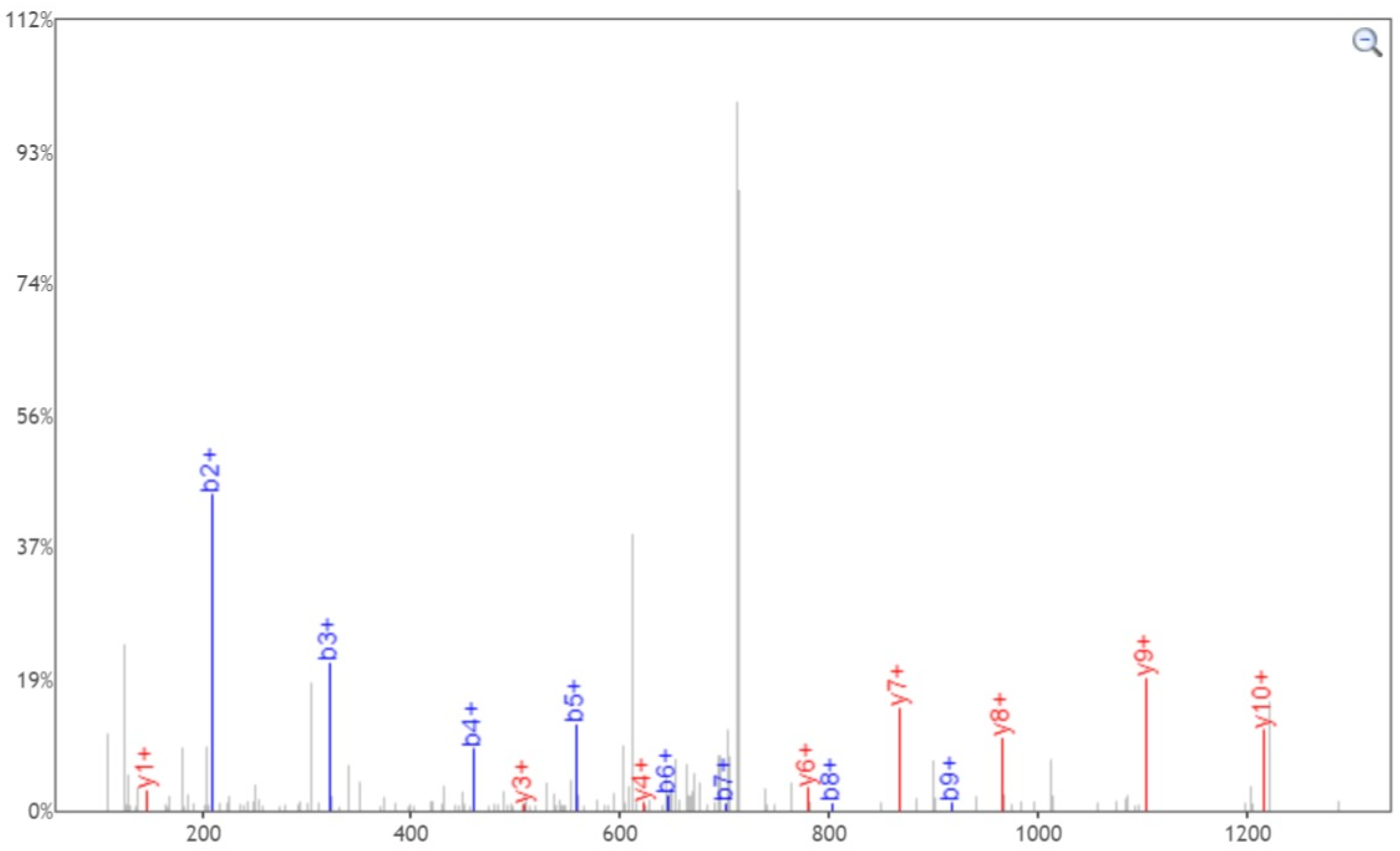

Click and drag in the plot to zoom X: Y: Zoom Out Print Enable tooltip

b+	#	Seq	#	y+
138.0662	1	H	12	
209.1033	2	A	11	1287.6539
322.1874	3	I	10	1216.6168
459.2463	4	H	9	1103.5327
558.3147	5	V	8	966.4738
645.3467	6	S	7	867.4054
702.3682	7	G	6	780.3734
803.4159	8	T	5	723.3519
917.4588	9	N	4	622.3042
974.4803	10	G	3	508.2613
1278.6073	11	T	2	451.2399
	12	K	1	147.1128

[Click] to move table

Static Modifications:
C: 57.02146

Variable Modifications:
T: 203.079373
