# Supplementary material for: Site-Specific O-Glycosylation Analysis of SARS-CoV-2 Spike Protein Produced in Insect and Human Cells
Source: Viruses. 2021 Mar 25;13(4):551. doi: 10.3390/v13040551 (PMC8064498; doi:10.3390/v13040551)
Supplement: Supplementary file 1 [file viruses-13-00551-s001.zip › Dataset S7. Spectra Q Exactive.zip!/spectrum list-Exp_2/T76-2-N.pdf]

Fragment Ion View

Project name: 20200330COV19
Sample name: 20200416_COV19-double_2
Created date: 2020-04-18 15:22:56.0
Search name:
Activation type: CID

Peptide sequence: F.HAIHVSGTN(2.988261)GTK.R
Scan number: 7968
Calculated M+H: 1224.6217
Measured M+H: 1224.6249
Charge state: 3
File name: 20200416_COV19-double_2
search type: light

Ions:
a 1+ 2+ 3+
b ☑1+ ☑2+ 3+
c 1+ 2+ 3+
x 1+ 2+ 3+
y ☑1+ ☑2+ 3+
z 1+ 2+ 3+
[Deselect All]

Neutral Loss:
- H_2O (o)
- NH_3 (*)
- H_3PO_4 (^)
- Precursor (#)
- Precursor with reduced charges

Mass Type: ◉Mono ○Avg

Mass Tol: 20

○Dalton ◉PPM

Update

Peak Assignment:
- ◉Most Intense
- ○Nearest Match
- Peak Detect
- Precursor Removal

Peak Labels: ◉Ion ○m/z ○None

Width: 750

Height: 450

HAIHVSGTNGTK, MH+ 1224.6217, m/z 408.8788
File: 20200416_COV19-double_2, Scan: 7968, Precursor m/z: 408.8798, Charge: 3

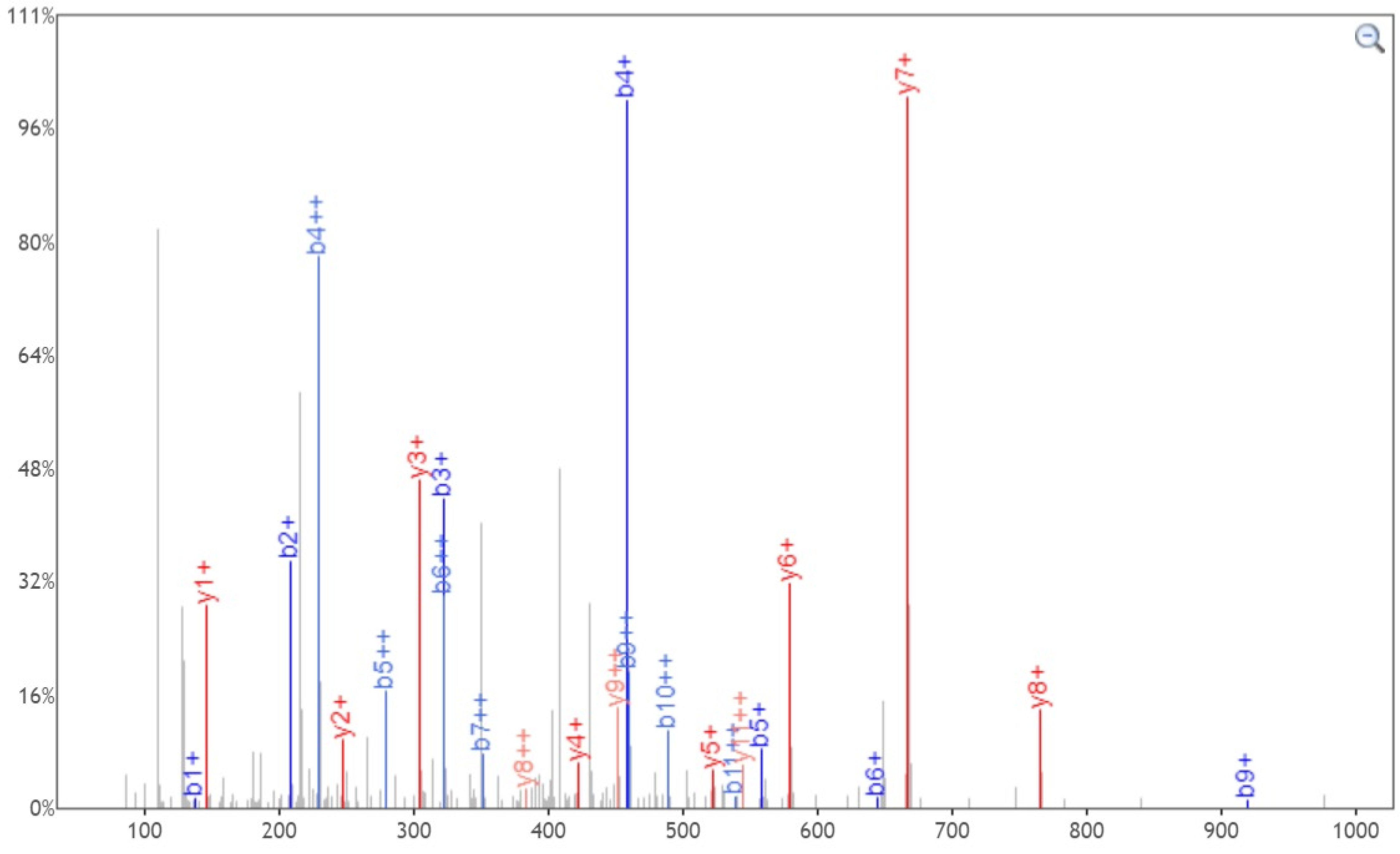

Click and drag in the plot to zoom X:☑ Y:☐ Zoom Out Print ☐Enable tooltip

b+	b2+	#	Seq	#	y+	y2+
138.0662	69.5367	1	H	12		
209.1033	105.0553	2	A	11	1087.5628	544.2850
322.1874	161.5973	3	I	10	1016.5257	508.7665
459.2463	230.1268	4	H	9	903.4416	452.2245
558.3147	279.6610	5	V	8	766.3827	383.6950
645.3467	323.1770	6	S	7	667.3143	334.1608
702.3682	351.6877	7	G	6	580.2823	290.6448
803.4159	402.2116	8	T	5	523.2608	262.1340
920.4471	460.7272	9	N	4	422.2131	211.6102
977.4685	489.2379	10	G	3	305.1819	153.0946
1078.5162	539.7617	11	T	2	248.1605	124.5839
		12	K	1	147.1128	74.0600

[Click] to move table

Static Modifications:
C: 57.02146

Variable Modifications:
N: 2.988261
