# Supplementary material for: Site-Specific O-Glycosylation Analysis of SARS-CoV-2 Spike Protein Produced in Insect and Human Cells
Source: Viruses. 2021 Mar 25;13(4):551. doi: 10.3390/v13040551 (PMC8064498; doi:10.3390/v13040551)
Supplement: Supplementary file 1 [file viruses-13-00551-s001.zip › Dataset S7. Spectra Q Exactive.zip!/spectrum list-Exp_2/S1097-1-Y.pdf]

Fragment Ion View

Project name: 20200330COV19
Sample name: 20200416_COV19-double_2
Created date: 2020-04-18 15:22:56.0
Search name:
Activation type: CID

Peptide sequence: R.EGVFVS(203.079373)NGTHW.F
Scan number: 44068
Calculated M+H: 1435.6488
Measured M+H: 1435.6523
Charge state: 2
File name: 20200416_COV19-double_2
search type: light

Ions:
a 1⁺ 2⁺ 3⁺
b 1⁺ 2⁺ 3⁺
c 1⁺ 2⁺ 3⁺
x 1⁺ 2⁺ 3⁺
y 1⁺ 2⁺ 3⁺
z 1⁺ 2⁺ 3⁺
[Deselect All]

Neutral Loss:
H_2O (o)
NH_3 (*)
H_3PO_4 (^)
Precursor (#)
Precursor with reduced charges

Mass Type:
Mono Avg

Mass Tol: 20
Dalton PPM

Update

Peak Assignment:
Most Intense
Nearest Match
Peak Detect
Precursor Removal

Peak Labels:
Ion m/z
None

Width: 750

Height: 450

EGVFVSNGTHW, MH+ 1435.6488, m/z 718.3281
File: 20200416_COV19-double_2, Scan: 44068, Precursor m/z: 718.32983, Charge: 2

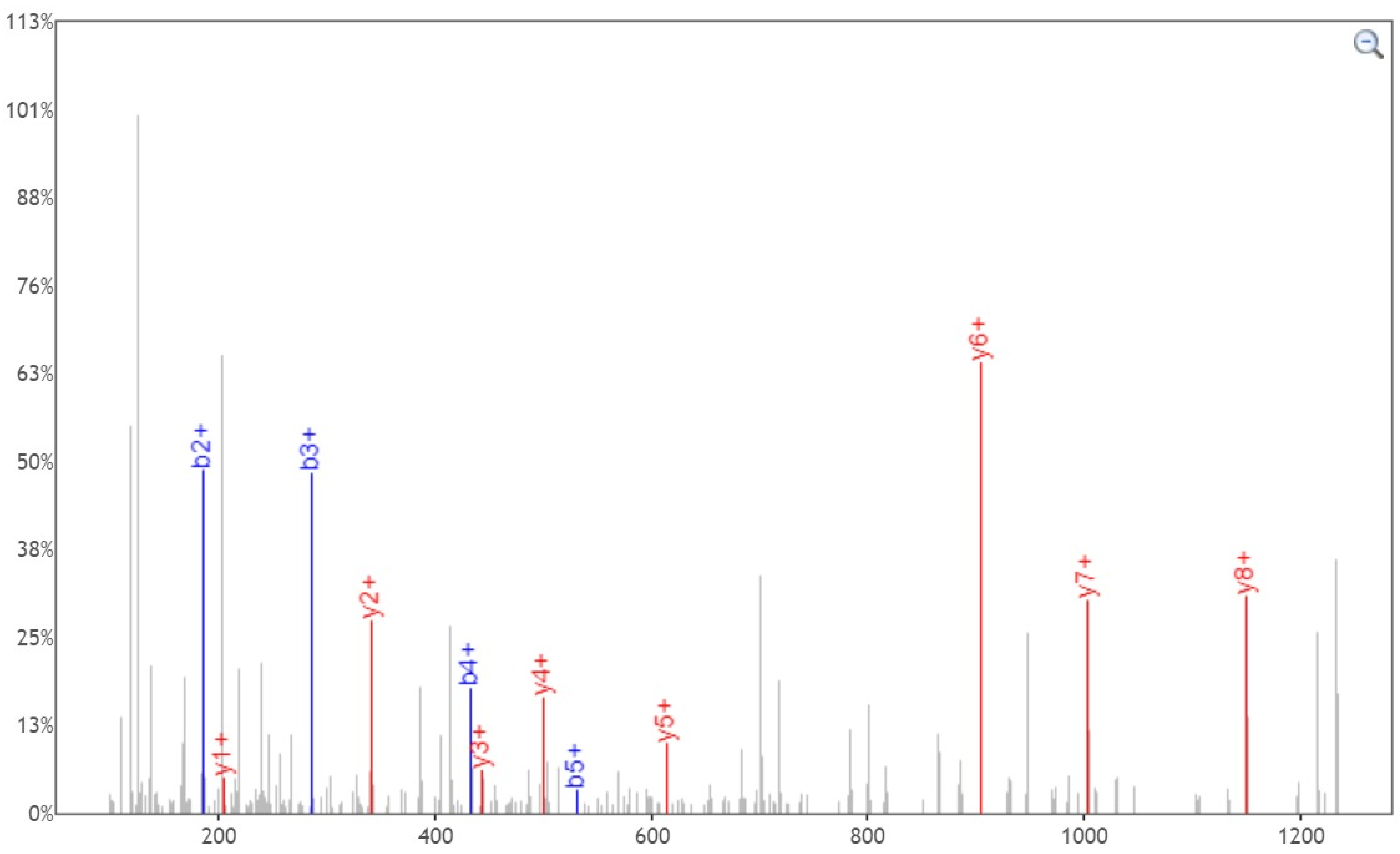

Click and drag in the plot to zoom X: Y: Zoom Out Print Enable tooltip

b+	#	Seq	#	y+
130.0499	1	E	11	
187.0713	2	G	10	1306.6062
286.1397	3	V	9	1249.5848
433.2082	4	F	8	1150.5164
532.2766	5	V	7	1003.4480
822.3880	6	S	6	904.3795
936.4309	7	N	5	614.2681
993.4524	8	G	4	500.2252
1094.5000	9	T	3	443.2037
1231.5590	10	H	2	342.1561
	11	W	1	205.0972

[Click] to move table

Static Modifications:
C: 57.02146

Variable Modifications:
S: 203.079373
