# Supplementary material for: Site-Specific O-Glycosylation Analysis of SARS-CoV-2 Spike Protein Produced in Insect and Human Cells
Source: Viruses. 2021 Mar 25;13(4):551. doi: 10.3390/v13040551 (PMC8064498; doi:10.3390/v13040551)
Supplement: Supplementary file 1 [file viruses-13-00551-s001.zip › Dataset S7. Spectra Q Exactive.zip!/spectrum list-Exp_2/S1097-2-Y.pdf]

Fragment Ion View

Project name: 20200330COV19
Sample name: 20200416_COV19-double_2
Created date: 2020-04-18 15:22:56.0
Search name:
Activation type: CID

Peptide sequence: R.EGVFVS(203.079373)NGTHWF.V
Scan number: 52170
Calculated M+H: 1582.7173
Measured M+H: 1582.7219
Charge state: 2
File name: 20200416_COV19-double_2
search type: light

Ions:
a 1+ 2+ 3+
b 1+ 2+ 3+
c 1+ 2+ 3+
x 1+ 2+ 3+
y 1+ 2+ 3+
z 1+ 2+ 3+
[Deselect All]

Neutral Loss:
H_2O (o)
NH_3 (*)
H_3PO_4 (^)
Precursor (#)
Precursor with reduced charges

Mass Type:
Mono Avg

Mass Tol: 20
Dalton PPM

Update

Peak Assignment:
Most Intense
Nearest Match
Peak Detect
Precursor Removal

Peak Labels:
Ion m/z
None

Width: 750

Height: 450

EGVFVSNGTHWF, MH+ 1582.7172, m/z 791.8623
File: 20200416_COV19-double_2, Scan: 52170, Precursor m/z: 791.8646, Charge: 2

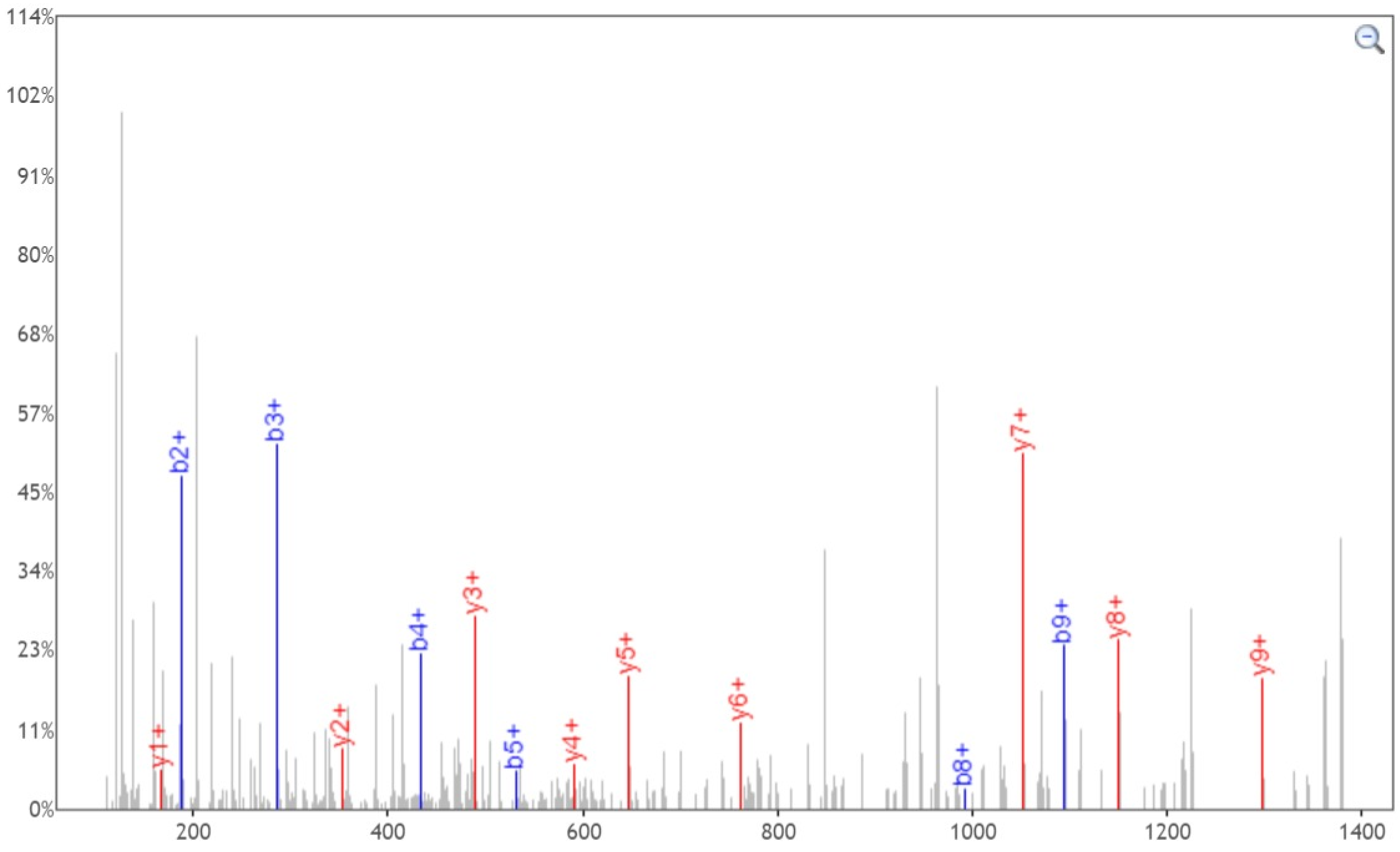

Click and drag in the plot to zoom X: Y: Zoom Out Print Enable tooltip

b+	#	Seq	#	y+
130.0499	1	E	12	
187.0713	2	G	11	1453.6747
286.1397	3	V	10	1396.6532
433.2082	4	F	9	1297.5848
532.2766	5	V	8	1150.5164
822.3880	6	S	7	1051.4480
936.4309	7	N	6	761.3365
993.4524	8	G	5	647.2936
1094.5000	9	T	4	590.2722
1231.5590	10	H	3	489.2245
1417.6383	11	W	2	352.1656
	12	F	1	166.0863

[Click] to move table

Static Modifications:
C: 57.02146

Variable Modifications:
S: 203.079373
