# Supplementary material for: Site-Specific O-Glycosylation Analysis of SARS-CoV-2 Spike Protein Produced in Insect and Human Cells
Source: Viruses. 2021 Mar 25;13(4):551. doi: 10.3390/v13040551 (PMC8064498; doi:10.3390/v13040551)
Supplement: Supplementary file 1 [file viruses-13-00551-s001.zip › Dataset S7. Spectra Q Exactive.zip!/spectrum list-Exp_2/T236-1-Y.pdf]

Fragment Ion View

Project name: 20200330COV19
Sample name: 20200416_COV19-double_2
Created date: 2020-04-18 15:22:56.0
Search name:
Activation type: CID

Peptide sequence: F.SALEPLVDLPIGINIT(203.079373).R
Scan number: 56315
Calculated M+H: 1868.0262
Measured M+H: 1868.0323
Charge state: 2
File name: 20200416_COV19-double_2
search type: light

Ions:

a ☐1+ ☐2+ ☐3+
b ☑1+ ☐2+ ☐3+
c ☐1+ ☐2+ ☐3+
x ☐1+ ☐2+ ☐3+
y ☑1+ ☐2+ ☐3+
z ☐1+ ☐2+ ☐3+

[Deselect All]

Neutral Loss:

☐ H_2O (o)
☐ NH_3 (*)
☐ H_3PO_4 (^)
☐ Precursor (#)
☐ Precursor with reduced charges

Mass Type:
◉Mono ○Avg

Mass Tol: 20
○Dalton ◉PPM

Update

Peak Assignment:
◉Most Intense
○Nearest Match
☐Peak Detect
☐Precursor Removal

Peak Labels:
◉Ion ○m/z
○None

Width: 750

Height: 450

SALEPLVDLPIGINIT, MH+ 1868.0263, m/z 934.5168
File: 20200416_COV19-double_2, Scan: 56315, Precursor m/z: 934.51984, Charge: 2

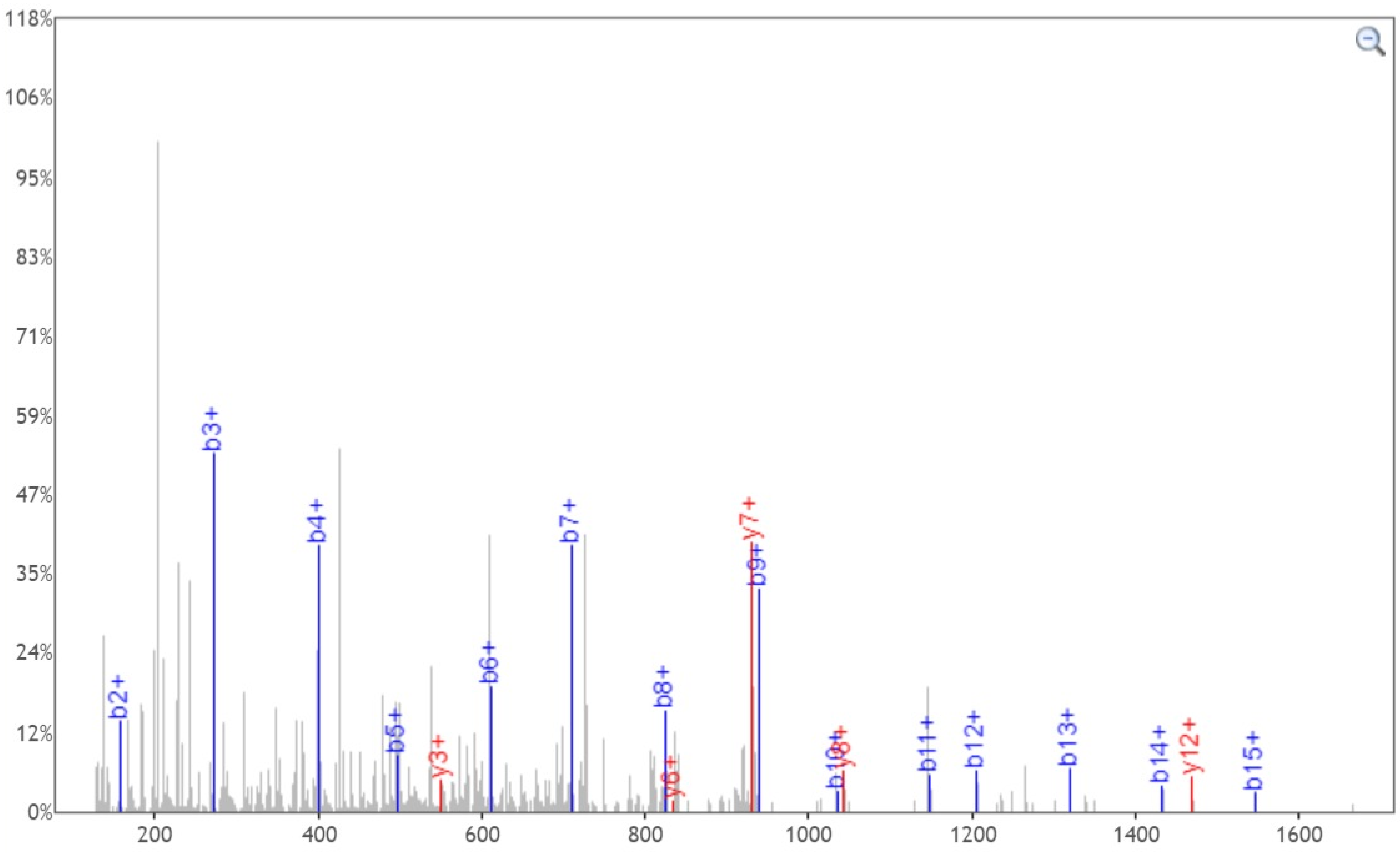

Click and drag in the plot to zoom X:☑ Y:☐ Zoom Out Print ☐Enable tooltip

b+	#	Seq	#	y+
88.0393	1	S	16	
159.0764	2	A	15	1780.9943
272.1605	3	L	14	1709.9571
401.2031	4	E	13	1596.8731
498.2558	5	P	12	1467.8305
611.3399	6	L	11	1370.7777
710.4083	7	V	10	1257.6937
825.4353	8	D	9	1158.6252
938.5193	9	L	8	1043.5983
1035.5721	10	P	7	930.5142
1148.6562	11	I	6	833.4615
1205.6776	12	G	5	720.3774
1318.7617	13	I	4	663.3559
1432.8046	14	N	3	550.2719
1545.8887	15	I	2	436.2290
	16	T	1	323.1449

[Click] to move table

Static Modifications:
C: 57.02146

Variable Modifications:
T: 203.079373
