# Supplementary material for: Site-Specific O-Glycosylation Analysis of SARS-CoV-2 Spike Protein Produced in Insect and Human Cells
Source: Viruses. 2021 Mar 25;13(4):551. doi: 10.3390/v13040551 (PMC8064498; doi:10.3390/v13040551)
Supplement: Supplementary file 1 [file viruses-13-00551-s001.zip › Dataset S7. Spectra Q Exactive.zip!/spectrum list-Exp_2/T63-1-N.pdf]

Fragment Ion View

Project name: 20200330COV19
Sample name: 20200416_COV19-double_2
Created date: 2020-04-18 15:22:56.0
Search name:
Activation type: CID

Peptide sequence: F.FSN(2.988261)VTWFHAIHVSGTN(2.988261)GTK.R
Scan number: 49970
Calculated M+H: 2109.017
Measured M+H: 2109.0225
Charge state: 3
File name: 20200416_COV19-double_2
search type: light

Ions:

a ☐1^+ ☐2^+ ☐3^+
b ☑1^+ ☑2^+ ☐3^+
c ☐1^+ ☐2^+ ☐3^+
x ☐1^+ ☐2^+ ☐3^+
y ☑1^+ ☑2^+ ☐3^+
z ☐1^+ ☐2^+ ☐3^+
[Deselect All]

Neutral Loss:

☐ H_2O (o)
☐ NH_3 (*)
☐ H_3PO_4 (^)
☐ Precursor (#)
☐ Precursor with reduced charges

Mass Type: ◉Mono ○Avg

Mass Tol: 20

○Dalton ◉PPM

Update

Peak Assignment:
◉Most Intense
○Nearest Match
☐Peak Detect
☐Precursor Removal

Peak Labels:
◉Ion ○m/z
○None

Width: 750

Height: 450

FSNVTWFHAIHVSGTNGTK, MH+ 2109.0172, m/z 703.6772
File: 20200416_COV19-double_2, Scan: 49970, Precursor m/z: 703.679, Charge: 3

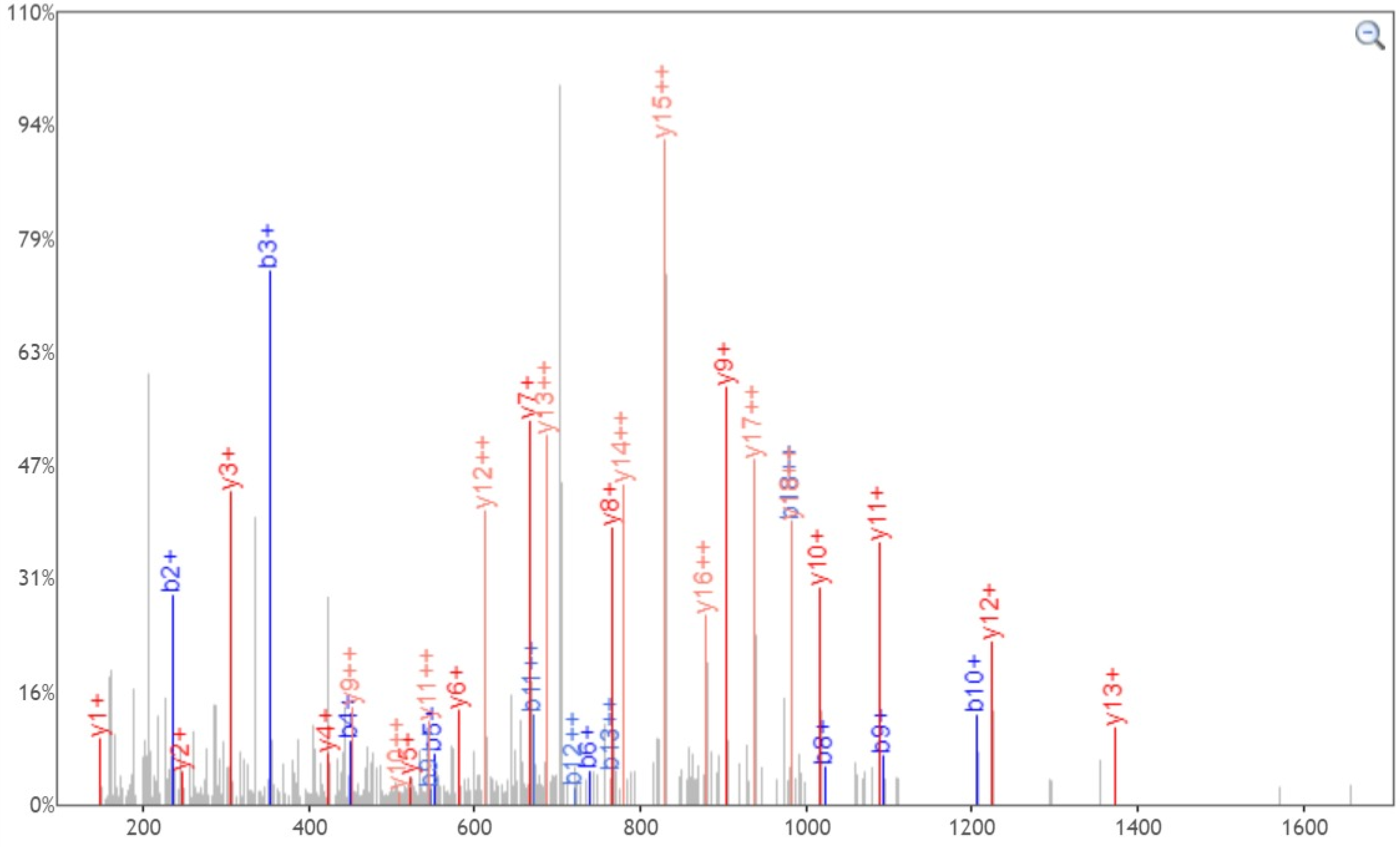

Click and drag in the plot to zoom X:☑ Y:☐ Zoom Out Print ☐Enable tooltip

b+	b2+	#	Seq	#	y+	y2+
148.0757	74.5415	1	F	19		
235.1077	118.0575	2	S	18	1961.9488	981.4780
352.1389	176.5731	3	N	17	1874.9167	937.9620
451.2073	226.1073	4	V	16	1757.8855	879.4464
552.2550	276.6311	5	T	15	1658.8171	829.9122
738.3343	369.6708	6	W	14	1557.7694	779.3884
885.4027	443.2050	7	F	13	1371.6901	686.3487
1022.4616	511.7345	8	H	12	1224.6217	612.8145
1093.4988	547.2530	9	A	11	1087.5628	544.2850
1206.5828	603.7950	10	I	10	1016.5257	508.7665
1343.6417	672.3245	11	H	9	903.4416	452.2245
1442.7101	721.8587	12	V	8	766.3827	383.6950
1529.7422	765.3747	13	S	7	667.3143	334.1608
1586.7636	793.8855	14	G	6	580.2823	290.6448
1687.8113	844.4093	15	T	5	523.2608	262.1340
1804.8425	902.9249	16	N	4	422.2131	211.6102
1861.8640	931.4356	17	G	3	305.1819	153.0946
1962.9116	981.9595	18	T	2	248.1605	124.5839
		19	K	1	147.1128	74.0600

[Click] to move table

Static Modifications:
C: 57.02146

Variable Modifications:
N: 2.988261
