# Supplementary material for: Site-Specific O-Glycosylation Analysis of SARS-CoV-2 Spike Protein Produced in Insect and Human Cells
Source: Viruses. 2021 Mar 25;13(4):551. doi: 10.3390/v13040551 (PMC8064498; doi:10.3390/v13040551)
Supplement: Supplementary file 1 [file viruses-13-00551-s001.zip › Dataset S7. Spectra Q Exactive.zip!/spectrum list-Exp_2/T63-1-Y.pdf]

Fragment Ion View

Project name: 20200330COV19
Sample name: 20200416_COV19-double_2
Created date: 2020-04-18 15:22:56.0
Search name:
Activation type: CID

Peptide sequence: F.FSNVT(203.079373)WFHAIHVSGTN(2.988261)GTK.R
Scan number: 47985
Calculated M+H: 2309.1084
Measured M+H: 2309.118
Charge state: 4
File name: 20200416_COV19-double_2
search type: light

Ions:
a 1+ 2+ 3+
b 1+ 2+ 3+
c 1+ 2+ 3+
x 1+ 2+ 3+
y 1+ 2+ 3+
z 1+ 2+ 3+
[Deselect All]

Neutral Loss:
H_2O (o)
NH_3 (*)
H_3PO_4 (^)
Precursor (#)
Precursor with reduced charges

Mass Type: Mono Avg

Mass Tol: 20
Dalton PPM

Update

Peak Assignment:
Most Intense
Nearest Match
Peak Detect
Precursor Removal

Peak Labels:
Ion m/z
None

Width: 750

Height: 450

FSNVTWFHAIHVSGTNGTK, MH+ 2309.1083, m/z 578.0325
File: 20200416_COV19-double_2, Scan: 47985, Precursor m/z: 578.0349, Charge: 4

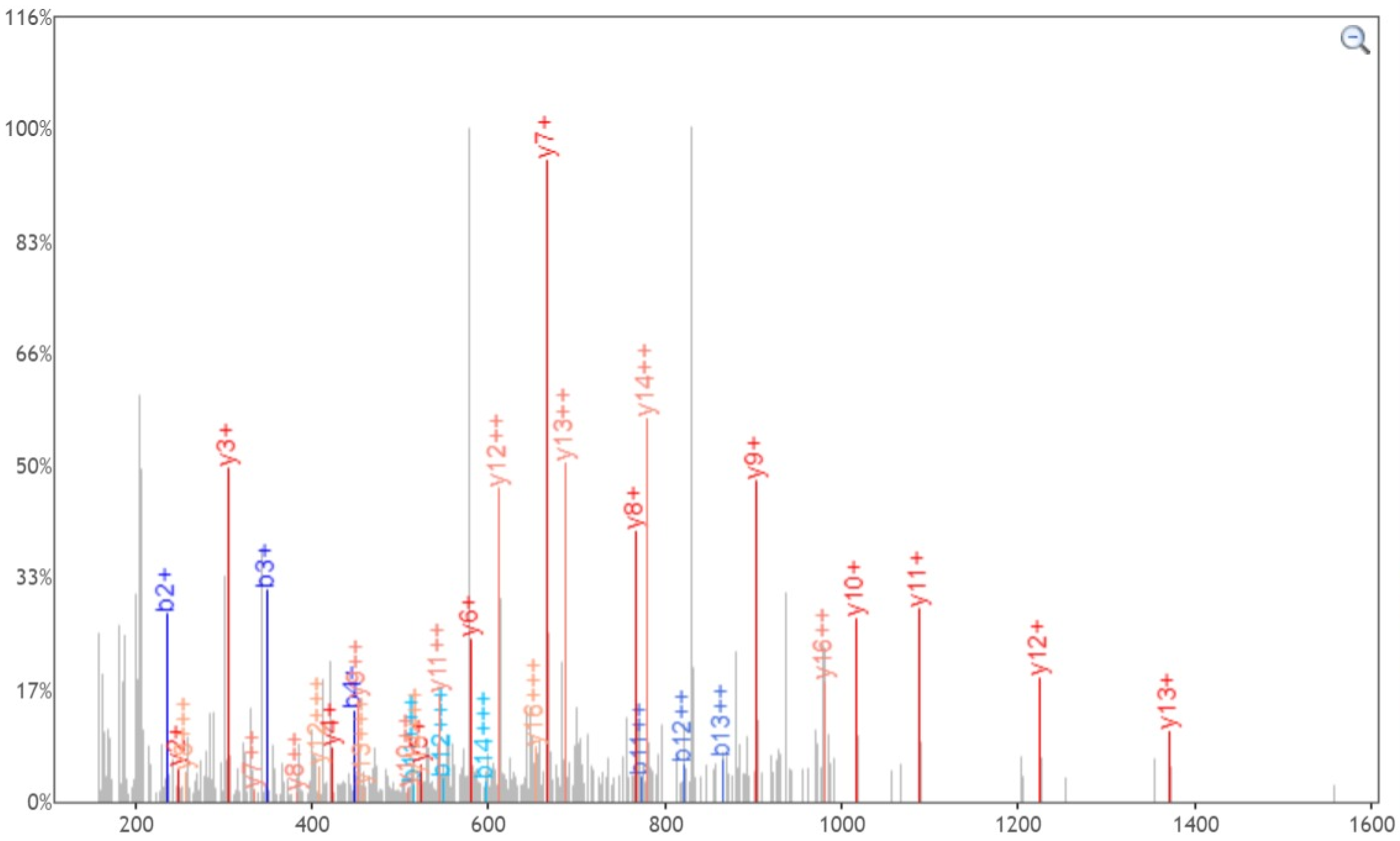

Click and drag in the plot to zoom X: Y: Zoom Out Print Enable tooltip

b+	b2+	b3+	#	Seq	#	y+	y2+	y3+
148.0757	74.5415	50.0301	1	F	19			
235.1077	118.0575	79.0408	2	S	18	2162.0399	1081.5236	721.3515
349.1506	175.0790	117.0551	3	N	17	2075.0078	1038.0076	692.3408
448.2191	224.6132	150.0779	4	V	16	1960.9649	980.9861	654.3265
752.3461	376.6767	251.4536	5	T	15	1861.8965	931.4519	621.3037
938.4254	469.7164	313.4800	6	W	14	1557.7694	779.3884	519.9280
1085.4938	543.2506	362.5028	7	F	13	1371.6901	686.3487	457.9016
1222.5528	611.7800	408.1891	8	H	12	1224.6217	612.8145	408.8788
1293.5899	647.2986	431.8681	9	A	11	1087.5628	544.2850	363.1925
1406.6739	703.8406	469.5628	10	I	10	1016.5257	508.7665	339.5134
1543.7328	772.3701	515.2491	11	H	9	903.4416	452.2245	301.8187
1642.8013	821.9043	548.2719	12	V	8	766.3827	383.6950	256.1324
1729.8333	865.4203	577.2826	13	S	7	667.3143	334.1608	223.1096
1786.8547	893.9310	596.2898	14	G	6	580.2823	290.6448	194.0989
1887.9024	944.4549	629.9723	15	T	5	523.2608	262.1340	175.0918
2004.9336	1002.9704	668.9827	16	N	4	422.2131	211.6102	141.4092
2061.9551	1031.4812	687.9899	17	G	3	305.1819	153.0946	102.3988
2163.0028	1082.0050	721.6724	18	T	2	248.1605	124.5839	83.3917
			19	K	1	147.1128	74.0600	49.7091

[Click] to move table

Static Modifications:
C: 57.02146

Variable Modifications:
T: 203.079373
N: 2.988261
