# Supplementary material for: Site-Specific O-Glycosylation Analysis of SARS-CoV-2 Spike Protein Produced in Insect and Human Cells
Source: Viruses. 2021 Mar 25;13(4):551. doi: 10.3390/v13040551 (PMC8064498; doi:10.3390/v13040551)
Supplement: Supplementary file 1 [file viruses-13-00551-s001.zip › Dataset S7. Spectra Q Exactive.zip!/spectrum list-Exp_2/T114-1-N.pdf]

Home Database Project Job status Edit Profile Cloud Manual Download Tools

home / project / experiment / search

Fragment Ion View

Project name: 20200330COV19
Sample name: 20200416_COV19-double_2
Created date: 2020-04-18 15:22:56.0
Search name:
Activation type: CID

Peptide sequence: K.TQSLLIVN(203.079373)NATNVVIK.V
Scan number: 49009
Calculated M+H: 1930.0856
Measured M+H: 1930.0911
Charge state: 2
File name: 20200416_COV19-double_2
search type: light

Ions:
c 1+ 2+ 3+
x 1+ 2+ 3+
y 1+ 2+ 3+
z 1+ 2+ 3+
[Deselect All]

Neutral Loss:
H_2O (o)
NH_3 (*)
H_3PO_4 (^)
Precursor (#)
Precursor with reduced charges

Mass Type:
Mono Avg

Mass Tol: 20
Dalton PPM

Update

Peak Assignment:
Most Intense
Nearest Match
Peak Detect
Precursor Removal

Peak Labels:
Ion m/z
None

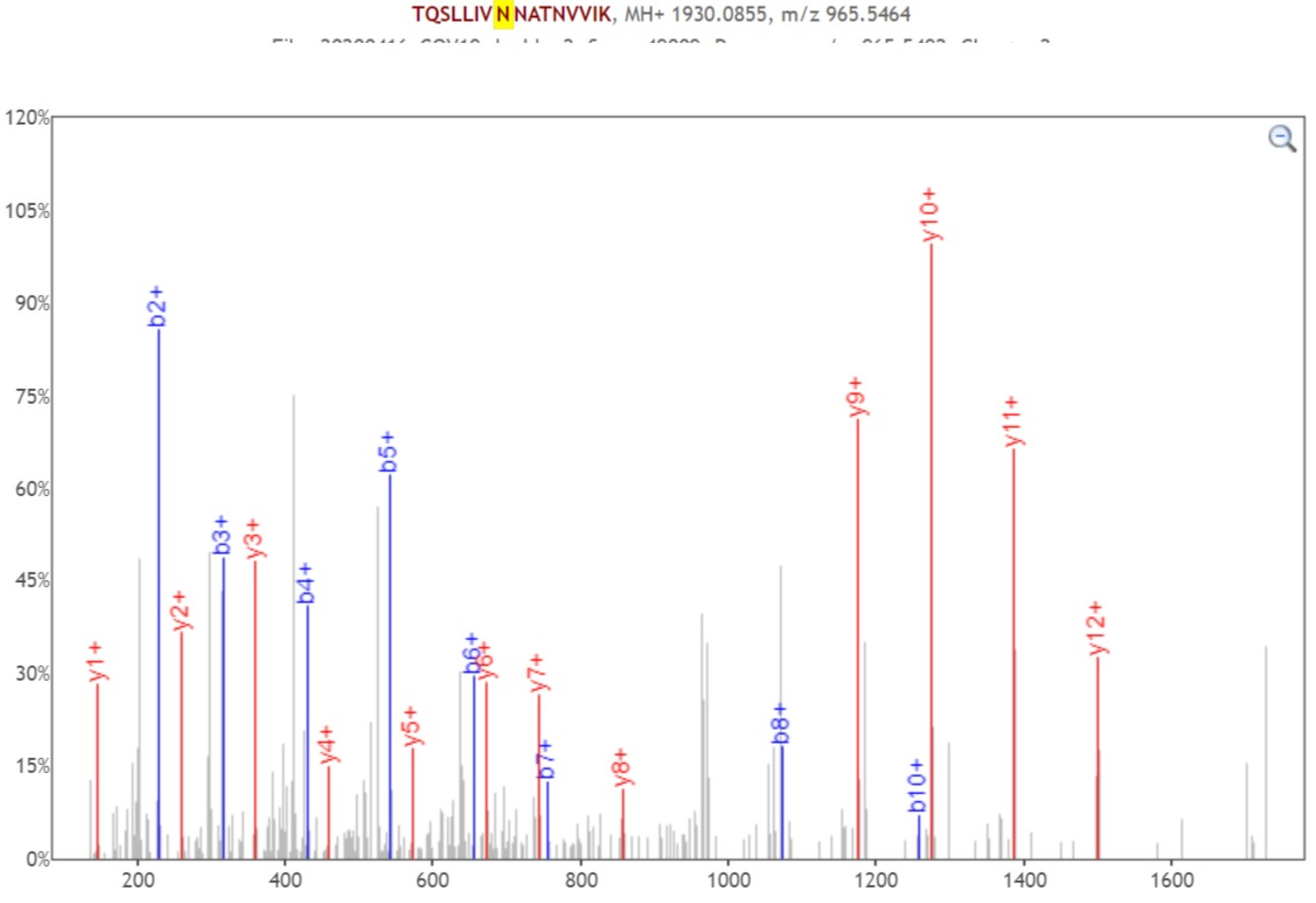

b+	#	Seq	#	y+
102.0550	1	T	16	
317.1456	3	S	14	1700.9793
430.2296	4	L	13	1613.9472
543.3137	5	L	12	1500.8632
656.3978	6	I	11	1387.7791
755.4662	7	V	10	1274.6951
1072.5885	8	N	9	1175.6266
1186.6314	9	N	8	858.5043
1257.6685	10	A	7	744.4614
1358.7162	11	T	6	673.4243
1472.7591	12	N	5	572.3766
1571.8275	13	V	4	458.3337
1670.8959	14	V	3	359.2653
1783.9800	15	I	2	260.1969
	16	K	1	147.1128

[Click] to move table

Static Modifications:
C: 57.02146

Variable Modifications:
N: 203.079373
