# Supplementary material for: Site-Specific O-Glycosylation Analysis of SARS-CoV-2 Spike Protein Produced in Insect and Human Cells
Source: Viruses. 2021 Mar 25;13(4):551. doi: 10.3390/v13040551 (PMC8064498; doi:10.3390/v13040551)
Supplement: Supplementary file 1 [file viruses-13-00551-s001.zip › Dataset S7. Spectra Q Exactive.zip!/spectrum list-Exp_2/T114-1-Y.pdf]

Fragment Ion View

Project name: 20200330COV19
Sample name: 20200416_COV19-double_2
Created date: 2020-04-18 15:22:56.0
Search name:
Activation type: CID

Peptide sequence: K.T(203.079373)QSLLIVNNATNVVIK.V
Scan number: 48968
Calculated M+H: 1930.0856
Measured M+H: 1930.0898
Charge state: 3
File name: 20200416_COV19-double_2
search type: light

Ions:
a 1+ 2+ 3+
b 1+ 2+ 3+
c 1+ 2+ 3+
x 1+ 2+ 3+
y 1+ 2+ 3+
z 1+ 2+ 3+
[Deselect All]

Neutral Loss:
H_2O (o)
NH_3 (*)
H_3PO_4 (^)
Precursor (#)
Precursor with reduced charges

Mass Type:
Mono Avg

Mass Tol: 20
Dalton PPM

Update

Peak Assignment:
Most Intense
Nearest Match
Peak Detect
Precursor Removal

Peak Labels:
Ion m/z
None

Width: 750

Height: 450

TQSLLIVNNATNVVIK, MH+ 1930.0855, m/z 644.0334
File: 20200416_COV19-double_2, Scan: 48968, Precursor m/z: 644.0348, Charge: 3

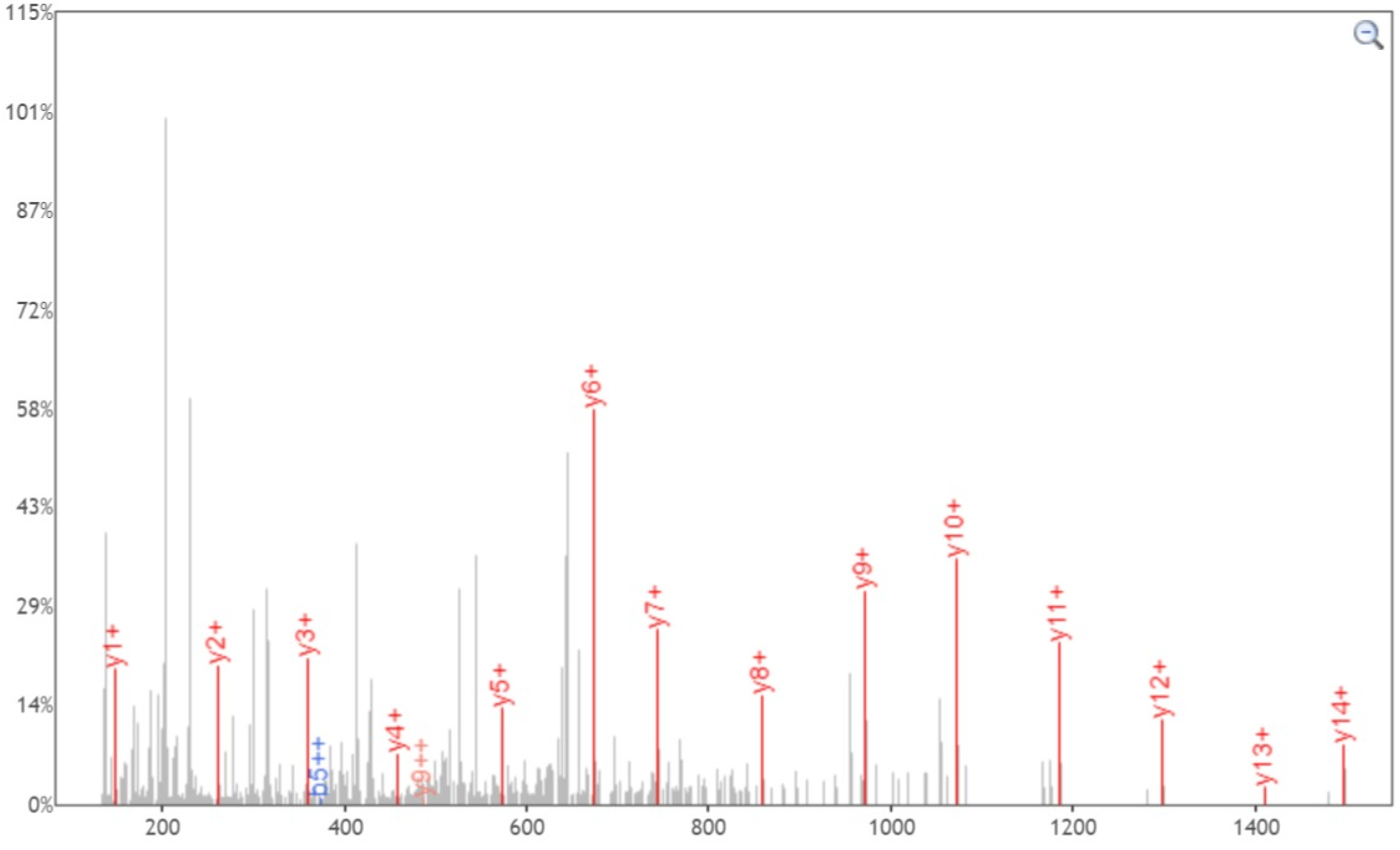

Click and drag in the plot to zoom X: Y: Zoom Out Print Enable tooltip

b+	b2+	#	Seq	#	y+	y2+
305.1343	153.0708	1	T	16		
433.1929	217.1001	2	Q	15	1625.9585	813.4829
520.2249	260.6161	3	S	14	1497.8999	749.4536
633.3090	317.1581	4	L	13	1410.8679	705.9376
746.3931	373.7002	5	L	12	1297.7838	649.3955
859.4771	430.2422	6	I	11	1184.6997	592.8535
958.5455	479.7764	7	V	10	1071.6157	536.3115
1072.5885	536.7979	8	N	9	972.5473	486.7773
1186.6314	593.8193	9	N	8	858.5043	429.7558
1257.6685	629.3379	10	A	7	744.4614	372.7343
1358.7162	679.8617	11	T	6	673.4243	337.2158
1472.7591	736.8832	12	N	5	572.3766	286.6919
1571.8275	786.4174	13	V	4	458.3337	229.6705
1670.8959	835.9516	14	V	3	359.2653	180.1363
1783.9800	892.4936	15	I	2	260.1969	130.6021
		16	K	1	147.1128	74.0600

[Click] to move table

Static Modifications:
C: 57.02146

Variable Modifications:
T: 203.079373
