# Supplementary material for: Site-Specific O-Glycosylation Analysis of SARS-CoV-2 Spike Protein Produced in Insect and Human Cells
Source: Viruses. 2021 Mar 25;13(4):551. doi: 10.3390/v13040551 (PMC8064498; doi:10.3390/v13040551)
Supplement: Supplementary file 1 [file viruses-13-00551-s001.zip › Dataset S7. Spectra Q Exactive.zip!/spectrum list-Exp_2/T167-1-Y.pdf]

Fragment Ion View

Project name: 20200330COV19
Sample name: 20200416_COV19-double_2
Created date: 2020-04-18 15:22:56.0
Search name:
Activation type: CID

Peptide sequence: R.VYSSANNCT(203.079373)FEYVSQPFLMDLEGK.Q
Scan number: 55875
Calculated M+H: 3002.3435
Measured M+H: 3002.3643
Charge state: 3
File name: 20200416_COV19-double_2
search type: light

Ions:
a ☐1+ ☐2+ ☐3+
b ☑1+ ☑2+ ☐3+
c ☐1+ ☐2+ ☐3+
x ☐1+ ☐2+ ☐3+
y ☑1+ ☑2+ ☐3+
z ☐1+ ☐2+ ☐3+
[Deselect All]

Neutral Loss:
☐ H_2O (o)
☐ NH_3 (*)
☐ H_3PO_4 (^)
☐ Precursor (#)
☐ Precursor with reduced charges

Mass Type: ◉Mono ○Avg

Mass Tol: 20

○Dalton ◉PPM

Update

Peak Assignment:
◉Most Intense
○Nearest Match
☐Peak Detect
☐Precursor Removal

Peak Labels:
◉Ion ○m/z
○None

Width: 750

Height: 450

VYSSANNCTFEYVSQPFLMDLEGK, MH+ 3002.3434, m/z 1001.4527
File: 20200416_COV19-double_2, Scan: 55875, Precursor m/z: 1001.4596, Charge: 3

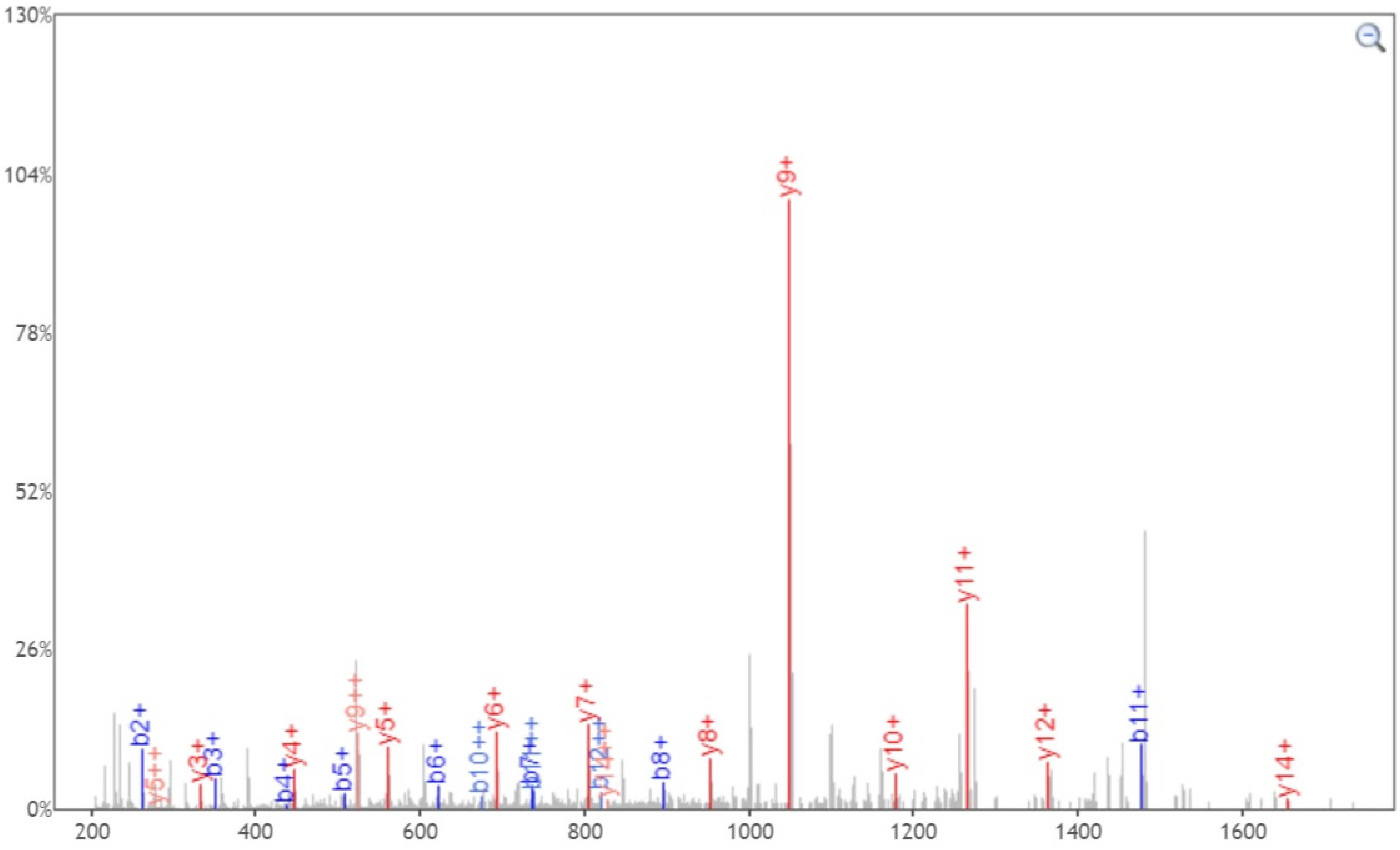

Click and drag in the plot to zoom X:☑ Y:☐ Zoom Out Print ☐Enable tooltip

b+	b2+	#	Seq	#	y+	y2+
100.0757	50.5415	1	V	24		
263.1390	132.0731	2	Y	23	2903.2750	1452.1411
350.1710	175.5892	3	S	22	2740.2117	1370.6095
437.2031	219.1052	4	S	21	2653.1796	1327.0935
508.2402	254.6237	5	A	20	2566.1476	1283.5774
622.2831	311.6452	6	N	19	2495.1105	1248.0589
736.3260	368.6667	7	N	18	2381.0676	1191.0374
896.3567	448.6820	8	C	17	2267.0246	1134.0160
1200.4837	600.7455	9	T	16	2106.9940	1054.0006
1347.5522	674.2797	10	F	15	1802.8669	901.9371
1476.5947	738.8010	11	E	14	1655.7985	828.4029
1639.6581	820.3327	12	Y	13	1526.7559	763.8816
1738.7265	869.8669	13	V	12	1363.6926	682.3499
1825.7585	913.3829	14	S	11	1264.6242	632.8157
1953.8171	977.4122	15	Q	10	1177.5922	589.2997
2050.8699	1025.9386	16	P	9	1049.5336	525.2704
2197.9383	1099.4728	17	F	8	952.4808	476.7441
2311.0223	1156.0148	18	L	7	805.4124	403.2098
2442.0628	1221.5350	19	M	6	692.3284	346.6678
2557.0898	1279.0485	20	D	5	561.2879	281.1476
2670.1738	1335.5906	21	L	4	446.2609	223.6341
2799.2164	1400.1118	22	E	3	333.1769	167.0921
2856.2379	1428.6226	23	G	2	204.1343	102.5708
		24	K	1	147.1128	74.0600

[Click] to move table

Static Modifications:
C: 57.02146

Variable Modifications:
T: 203.079373
