# Supplementary material for: Site-Specific O-Glycosylation Analysis of SARS-CoV-2 Spike Protein Produced in Insect and Human Cells
Source: Viruses. 2021 Mar 25;13(4):551. doi: 10.3390/v13040551 (PMC8064498; doi:10.3390/v13040551)
Supplement: Supplementary file 1 [file viruses-13-00551-s001.zip › Dataset S7. Spectra Q Exactive.zip!/spectrum list-Exp_1/S803-1-Y.pdf]

Fragment Ion View

Project name: 20200330COV19
Sample name: 20200416_COV19-double_1
Created date: 2020-04-18 15:22:56.0
Search name:
Activation type: CID

Peptide sequence: K.DFGGFNFS(203.079373)QILPDPSK.P
Scan number: 55714
Calculated M+H: 1971.9335
Measured M+H: 1971.9445
Charge state: 2
File name: 20200416_COV19-double_1
search type: light

Ions:
a [ ] 1+ [ ] 2+ [ ] 3+
b [x] 1+ [ ] 2+ [ ] 3+
c [ ] 1+ [ ] 2+ [ ] 3+
x [ ] 1+ [ ] 2+ [ ] 3+
y [x] 1+ [ ] 2+ [ ] 3+
z [ ] 1+ [ ] 2+ [ ] 3+
[Deselect All]

Neutral Loss:
[ ] H_2O (o)
[ ] NH_3 (*)
[ ] H_3PO_4 (^)
[ ] Precursor (#)
[ ] Precursor with reduced charges

Mass Type: (•) Mono ( ) Avg

Mass Tol: 20
( ) Dalton (•) PPM

Update

Peak Assignment:
(•) Most Intense
( ) Nearest Match
[ ] Peak Detect
[ ] Precursor Removal

Peak Labels:
(•) Ion ( ) m/z
( ) None

Width: 750

Height: 450

DFGGFNFSQILPDPSK, MH+ 1971.9334, m/z 986.4704
File: 20200416_COV19-double_1, Scan: 55714, Precursor m/z: 986.4759, Charge: 2

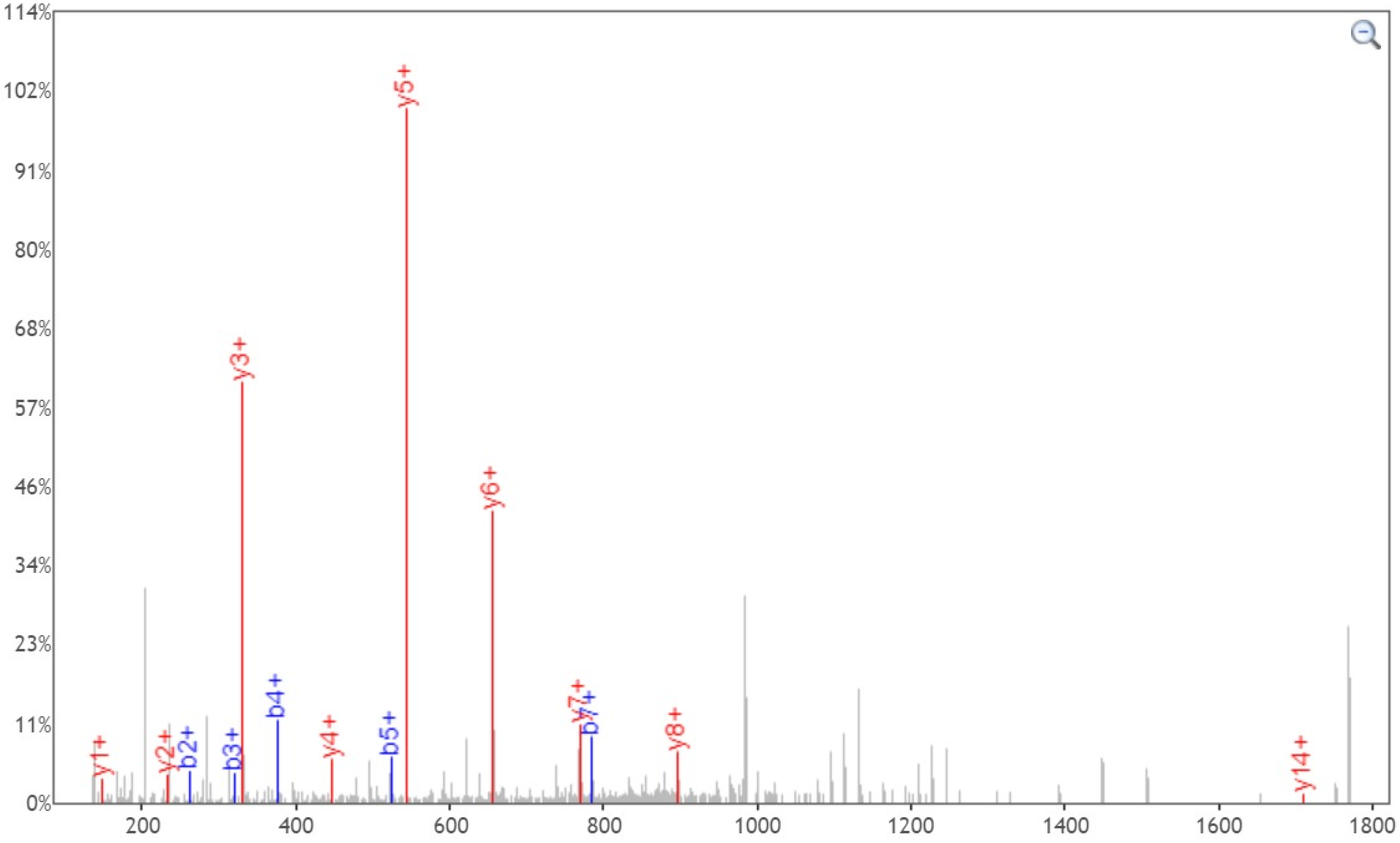

Click and drag in the plot to zoom X: [x] Y: [ ] Zoom Out Print [ ] Enable tooltip

b+	#	Seq	#	y+
116.0342	1	D	16	
263.1026	2	F	15	1856.9065
320.1241	3	G	14	1709.8381
377.1456	4	G	13	1652.8166
524.2140	5	F	12	1595.7952
638.2569	6	N	11	1448.7268
785.3253	7	F	10	1334.6838
1075.4367	8	S	9	1187.6154
1203.4953	9	Q	8	897.5040
1316.5794	10	I	7	769.4454
1429.6634	11	L	6	656.3614
1526.7162	12	P	5	543.2773
1641.7431	13	D	4	446.2245
1738.7959	14	P	3	331.1976
1825.8279	15	S	2	234.1448
	16	K	1	147.1128

[Click] to move table

Static Modifications:
C: 57.02146

Variable Modifications:
S: 203.079373
