# Supplementary material for: Site-Specific O-Glycosylation Analysis of SARS-CoV-2 Spike Protein Produced in Insect and Human Cells
Source: Viruses. 2021 Mar 25;13(4):551. doi: 10.3390/v13040551 (PMC8064498; doi:10.3390/v13040551)
Supplement: Supplementary file 1 [file viruses-13-00551-s001.zip › Dataset S7. Spectra Q Exactive.zip!/spectrum list-Exp_1/S803-1-N.pdf]

Fragment Ion View

Project name: 20200330COV19
Sample name: 20200416_COV19-double_1
Created date: 2020-04-18 15:22:56.0
Search name:
Activation type: CID

Peptide sequence: K.DFGGFN(2.988261)FSQILPDPSK.P
Scan number: 55942
Calculated M+H: 1771.8424
Measured M+H: 1771.8472
Charge state: 2
File name: 20200416_COV19-double_1
search type: light

Ions:
a ☐1+ ☐2+ ☐3+
b ☑1+ ☐2+ ☐3+
c ☐1+ ☐2+ ☐3+
x ☐1+ ☐2+ ☐3+
y ☑1+ ☐2+ ☐3+
z ☐1+ ☐2+ ☐3+
[Deselect All]

Neutral Loss:
☐ H_2O (o)
☐ NH_3 (*)
☐ H_3PO_4 (^)
☐ Precursor (#)
☐ Precursor with reduced charges

Mass Type:
◉Mono ○Avg

Mass Tol: 20
○Dalton ◉PPM

Update

Peak Assignment:
◉Most Intense
○Nearest Match
☐Peak Detect
☐Precursor Removal

Peak Labels:
◉Ion ○m/z
○None

Width: 750
Height: 450

DFGGFNFSQILPDPSK, MH+ 1771.8423, m/z 886.4248
File: 20200416_COV19-double_1, Scan: 55942, Precursor m/z: 886.42725, Charge: 2

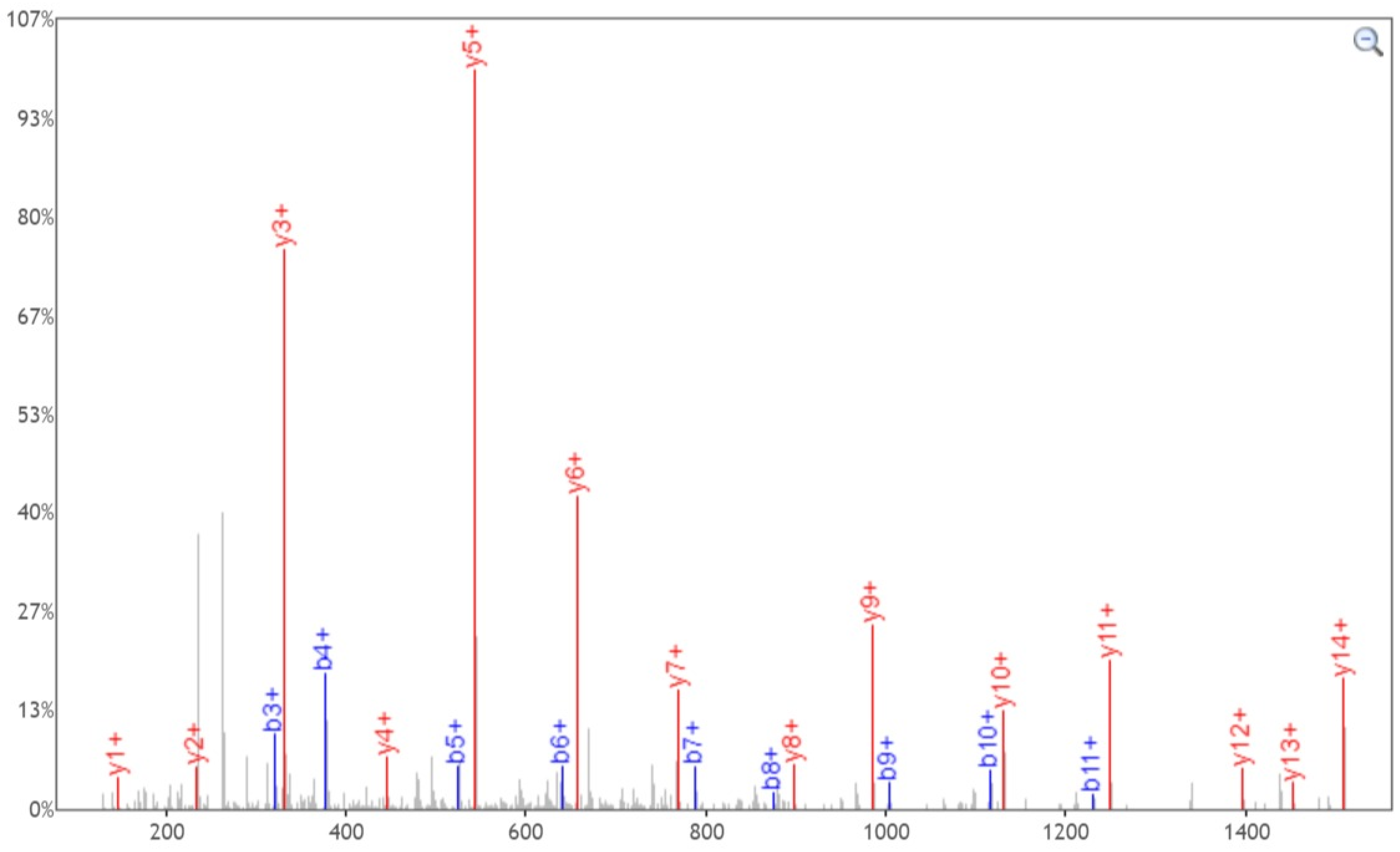

Click and drag in the plot to zoom X:☑ Y:☐ Zoom Out Print ☐Enable tooltip

b+	#	Seq	#	y+
116.0342	1	D	16	
263.1026	2	F	15	1656.8154
320.1241	3	G	14	1509.7470
377.1456	4	G	13	1452.7255
524.2140	5	F	12	1395.7041
641.2452	6	N	11	1248.6356
788.3136	7	F	10	1131.6045
875.3456	8	S	9	984.5360
1003.4042	9	Q	8	897.5040
1116.4882	10	I	7	769.4454
1229.5723	11	L	6	656.3614
1326.6251	12	P	5	543.2773
1441.6520	13	D	4	446.2245
1538.7048	14	P	3	331.1976
1625.7368	15	S	2	234.1448
	16	K	1	147.1128

[Click] to move table

Static Modifications:
C: 57.02146

Variable Modifications:
N: 2.988261
