# Supplementary material for: Site-Specific O-Glycosylation Analysis of SARS-CoV-2 Spike Protein Produced in Insect and Human Cells
Source: Viruses. 2021 Mar 25;13(4):551. doi: 10.3390/v13040551 (PMC8064498; doi:10.3390/v13040551)
Supplement: Supplementary file 1 [file viruses-13-00551-s001.zip › Dataset S7. Spectra Q Exactive.zip!/spectrum list-Exp_1/S1097-1-N.pdf]

Fragment Ion View

Project name: 20200330COV19
Sample name: 20200416_COV19-double_1
Created date: 2020-04-18 15:22:56.0
Search name:
Activation type: CID

Peptide sequence: R.EGVFVSN(2.988261)GTHW.F
Scan number: 47225
Calculated M+H: 1235.5577
Measured M+H: 1235.5635
Charge state: 2
File name: 20200416_COV19-double_1
search type: light

Ions:
a 1+ 2+ 3+
b 1+ 2+ 3+
c 1+ 2+ 3+
x 1+ 2+ 3+
y 1+ 2+ 3+
z 1+ 2+ 3+
[Deselect All]

Neutral Loss:
H_2O (o)
NH_3 (*)
H_3PO_4 (^)
Precursor (#)
Precursor with reduced charges

Mass Type:
Mono Avg

Mass Tol: 20
Dalton PPM

Update

Peak Assignment:
Most Intense
Nearest Match
Peak Detect
Precursor Removal

Peak Labels:
Ion m/z
None

Width: 750

Height: 450

EGVFVSNGTHW, MH+ 1235.5577, m/z 618.2825
File: 20200416_COV19-double_1, Scan: 47225, Precursor m/z: 618.2854, Charge: 2

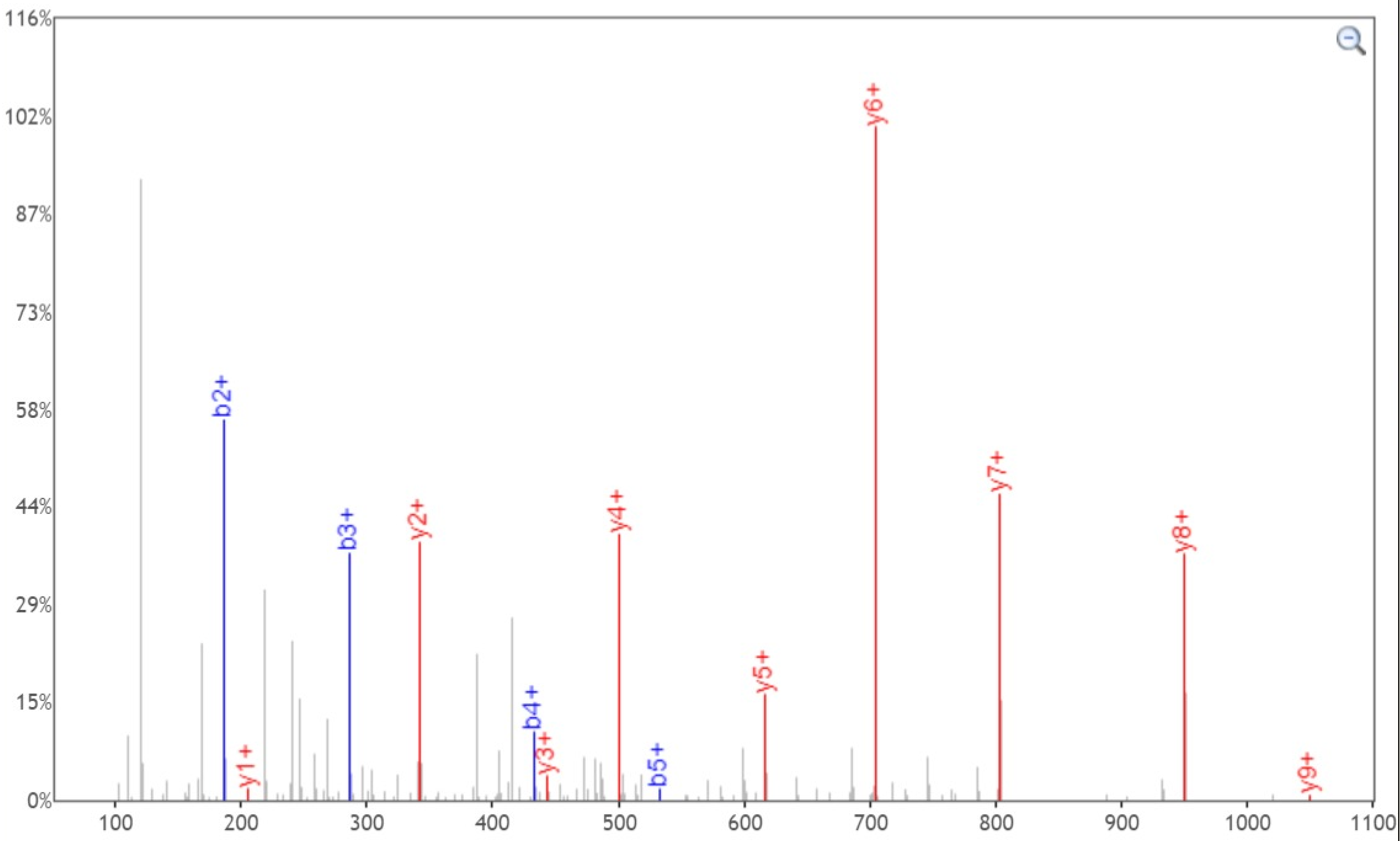

Click and drag in the plot to zoom X: Y: Zoom Out Print Enable tooltip

b+	#	Seq	#	y+
130.0499	1	E	11	
187.0713	2	G	10	1106.5151
286.1397	3	V	9	1049.4937
433.2082	4	F	8	950.4253
532.2766	5	V	7	803.3568
619.3086	6	S	6	704.2884
736.3398	7	N	5	617.2564
793.3613	8	G	4	500.2252
894.4089	9	T	3	443.2037
1031.4678	10	H	2	342.1561
	11	W	1	205.0972

[Click] to move table

Static Modifications:
C: 57.02146

Variable Modifications:
N: 2.988261
