# Supplementary material for: Site-Specific O-Glycosylation Analysis of SARS-CoV-2 Spike Protein Produced in Insect and Human Cells
Source: Viruses. 2021 Mar 25;13(4):551. doi: 10.3390/v13040551 (PMC8064498; doi:10.3390/v13040551)
Supplement: Supplementary file 1 [file viruses-13-00551-s001.zip › Dataset S7. Spectra Q Exactive.zip!/spectrum list-Exp_1/T63-2-Y.pdf]

Fragment Ion View

Project name: 20200330COV19
Sample name: 20200416_COV19-double_1
Created date: 2020-04-18 15:22:56.0
Search name:
Activation type: CID

Peptide sequence: R.SSVLHSTQDLFLPFFSNVT(203.079373)W.F
Scan number: 56688
Calculated M+H: 2528.2344
Measured M+H: 2528.2502
Charge state: 2
File name: 20200416_COV19-double_1
search type: light

Ions:
a ☐1+ ☐2+ ☐3+
b ☑1+ ☐2+ ☐3+
c ☐1+ ☐2+ ☐3+
x ☐1+ ☐2+ ☐3+
y ☑1+ ☐2+ ☐3+
z ☐1+ ☐2+ ☐3+
[Deselect All]

Neutral Loss:
☐ H_2O (o)
☐ NH_3 (*)
☐ H_3PO_4 (^)
☐ Precursor (#)
☐ Precursor with reduced charges

Mass Type:
◉Mono ○Avg

Mass Tol: 20
○Dalton ◉PPM

Update

Peak Assignment:
◉Most Intense
○Nearest Match
☐Peak Detect
☐Precursor Removal

Peak Labels:
◉Ion ○m/z
○None

Width: 750

Height: 450

SSVLHSTQDLFLPFFSNVTW, MH+ 2528.2344, m/z 1264.6208
File: 20200416_COV19-double_1, Scan: 56688, Precursor m/z: 1264.6288, Charge: 2

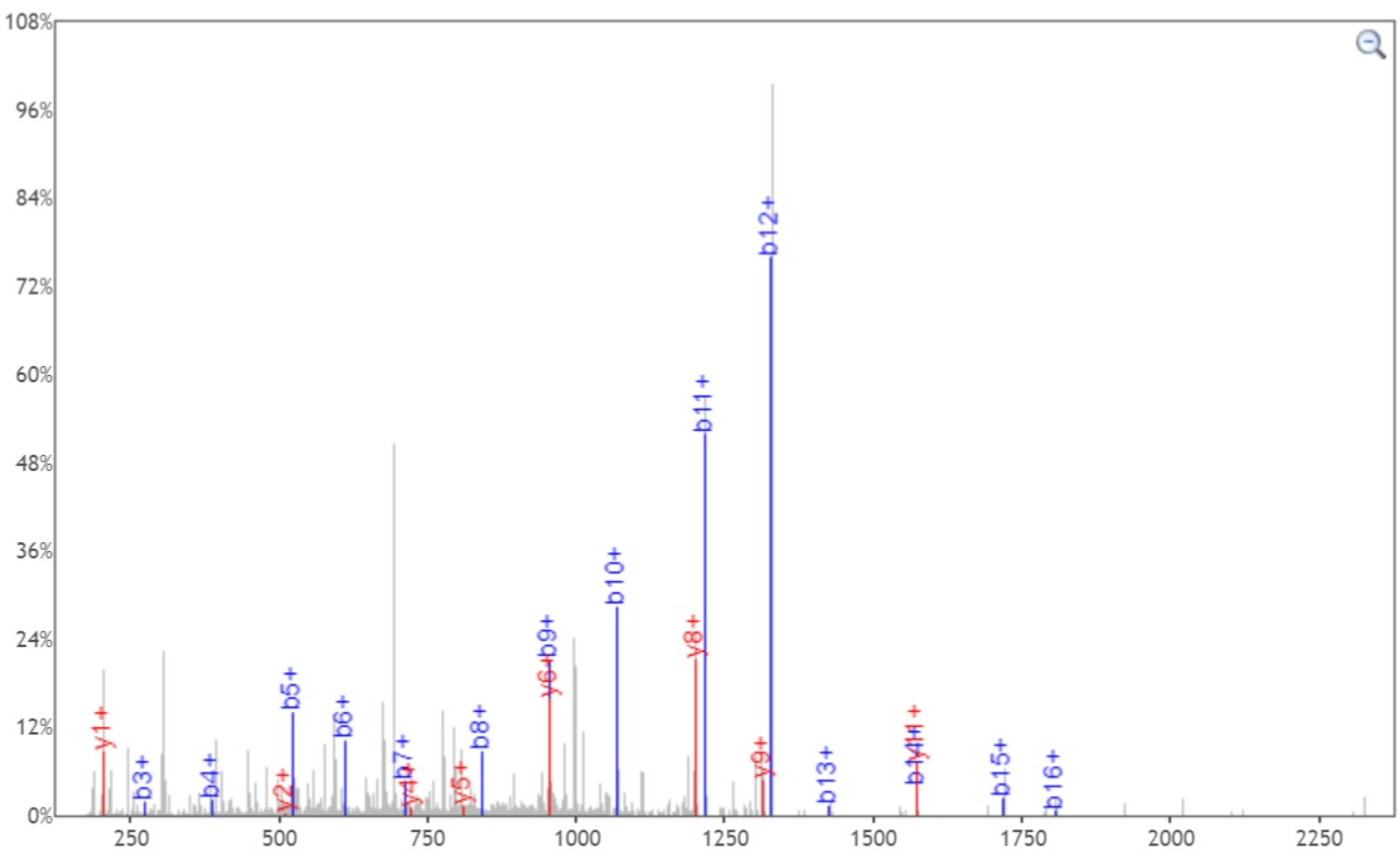

Click and drag in the plot to zoom X:☑ Y:☐ Zoom Out Print ☐Enable tooltip

b+	#	Seq	#	y+
88.0393	1	S	20	
175.0713	2	S	19	2441.2024
274.1397	3	V	18	2354.1703
387.2238	4	L	17	2255.1019
524.2827	5	H	16	2142.0178
611.3148	6	S	15	2004.9589
712.3624	7	T	14	1917.9269
840.4210	8	Q	13	1816.8792
955.4480	9	D	12	1688.8207
1068.5320	10	L	11	1573.7937
1215.6004	11	F	10	1460.7096
1328.6845	12	L	9	1313.6412
1425.7373	13	P	8	1200.5572
1572.8057	14	F	7	1103.5044
1719.8741	15	F	6	956.4360
1806.9061	16	S	5	809.3676
1920.9490	17	N	4	722.3355
2020.0175	18	V	3	608.2926
2324.1445	19	T	2	509.2242
	20	W	1	205.0972

[Click] to move table

Static Modifications:
C: 57.02146

Variable Modifications:
T: 203.079373
