# Supplementary material for: Site-Specific O-Glycosylation Analysis of SARS-CoV-2 Spike Protein Produced in Insect and Human Cells
Source: Viruses. 2021 Mar 25;13(4):551. doi: 10.3390/v13040551 (PMC8064498; doi:10.3390/v13040551)
Supplement: Supplementary file 1 [file viruses-13-00551-s001.zip › Dataset S7. Spectra Q Exactive.zip!/spectrum list-Exp_1/T63-2-N.pdf]

Fragment Ion View

Project name: 20200330COV19
Sample name: 20200416_COV19-double_1
Created date: 2020-04-18 15:22:56.0
Search name:
Activation type: CID

Peptide sequence: R.SSVLHSTQDLFLPFFSN(2.988261)VTW.F
Scan number: 56809
Calculated M+H: 2328.1433
Measured M+H: 2328.1624
Charge state: 2
File name: 20200416_COV19-double_1
search type: light

Ions:
a ☐1⁺ ☐2⁺ ☐3⁺
b ☑1⁺ ☐2⁺ ☐3⁺
c ☐1⁺ ☐2⁺ ☐3⁺
x ☐1⁺ ☐2⁺ ☐3⁺
y ☑1⁺ ☐2⁺ ☐3⁺
z ☐1⁺ ☐2⁺ ☐3⁺
[Deselect All]

Neutral Loss:
☐ H_2O (o)
☐ NH_3 (*)
☐ H_3PO_4 (^)
☐ Precursor (#)
☐ Precursor with reduced charges

Mass Type:
◉ Mono ○ Avg

Mass Tol: 20
○ Dalton ◉ PPM

Update

Peak Assignment:
◉ Most Intense
○ Nearest Match
☐ Peak Detect
☐ Precursor Removal

Peak Labels:
◉ Ion ○ m/z
○ None

Width: 750

Height: 450

SSVLHSTQDLFLPFFSNVTW, MH+ 2328.1433, m/z 1164.5753
File: 20200416_COV19-double_1, Scan: 56809, Precursor m/z: 1164.5848, Charge: 2

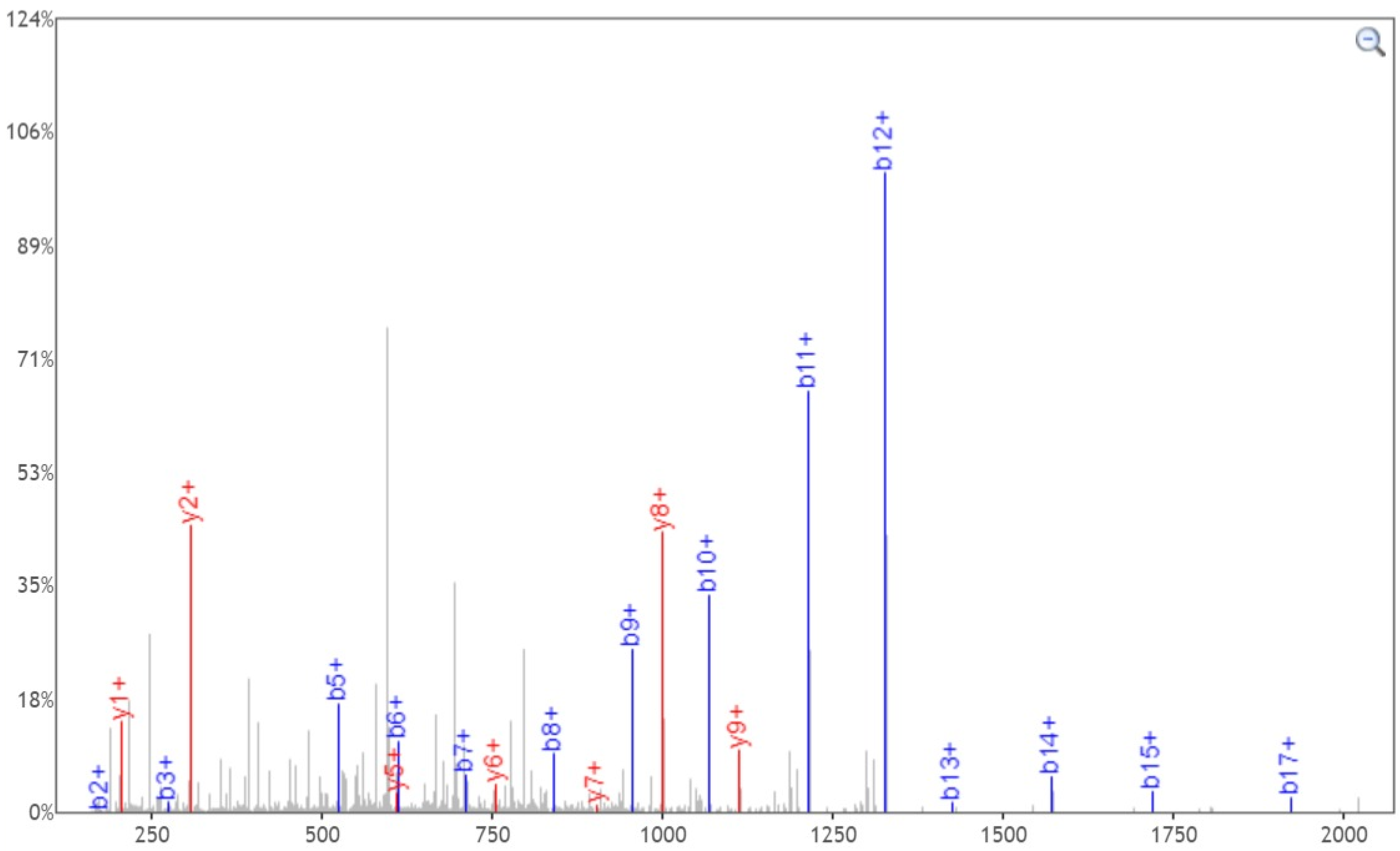

Click and drag in the plot to zoom X:☑ Y:☐ Zoom Out Print ☐Enable tooltip

b+	#	Seq	#	y+
88.0393	1	S	20	
175.0713	2	S	19	2241.1112
274.1397	3	V	18	2154.0792
387.2238	4	L	17	2055.0108
524.2827	5	H	16	1941.9267
611.3148	6	S	15	1804.8678
712.3624	7	T	14	1717.8358
840.4210	8	Q	13	1616.7881
955.4480	9	D	12	1488.7295
1068.5320	10	L	11	1373.7026
1215.6004	11	F	10	1260.6185
1328.6845	12	L	9	1113.5501
1425.7373	13	P	8	1000.4661
1572.8057	14	F	7	903.4133
1719.8741	15	F	6	756.3449
1806.9061	16	S	5	609.2765
1923.9373	17	N	4	522.2444
2023.0057	18	V	3	405.2132
2124.0534	19	T	2	306.1448
	20	W	1	205.0972

[Click] to move table

Static Modifications:
C: 57.02146

Variable Modifications:
N: 2.988261
