# Supplementary material for: Site-Specific O-Glycosylation Analysis of SARS-CoV-2 Spike Protein Produced in Insect and Human Cells
Source: Viruses. 2021 Mar 25;13(4):551. doi: 10.3390/v13040551 (PMC8064498; doi:10.3390/v13040551)
Supplement: Supplementary file 1 [file viruses-13-00551-s001.zip › Dataset S7. Spectra Q Exactive.zip!/spectrum list-Exp_1/T63-3-Y.pdf]

Fragment Ion View

Project name: 20200330COV19
Sample name: 20200416_COV19-double_1
Created date: 2020-04-18 15:22:56.0
Search name:
Activation type: CID

Peptide sequence: R.SSVLHSTQDLFLPFFSNVT(203.079373)WF.H
Scan number: 56819
Calculated M+H: 2675.3027
Measured M+H: 2675.312
Charge state: 2
File name: 20200416_COV19-double_1
search type: light

Ions:
a 1+ 2+ 3+
b 1+ 2+ 3+
c 1+ 2+ 3+
x 1+ 2+ 3+
y 1+ 2+ 3+
z 1+ 2+ 3+
[Deselect All]

Neutral Loss:
H_2O (o)
NH_3 (*)
H_3PO_4 (^)
Precursor (#)
Precursor with reduced charges

Mass Type:
Mono Avg

Mass Tol: 20
Dalton PPM

Update

Peak Assignment:
Most Intense
Nearest Match
Peak Detect
Precursor Removal

Peak Labels:
Ion m/z
None

Width: 750

Height: 450

SSVLHSTQDLFLPFFSNVTWF, MH+ 2675.3028, m/z 1338.1550
File: 20200416_COV19-double_1, Scan: 56819, Precursor m/z: 1338.1597, Charge: 2

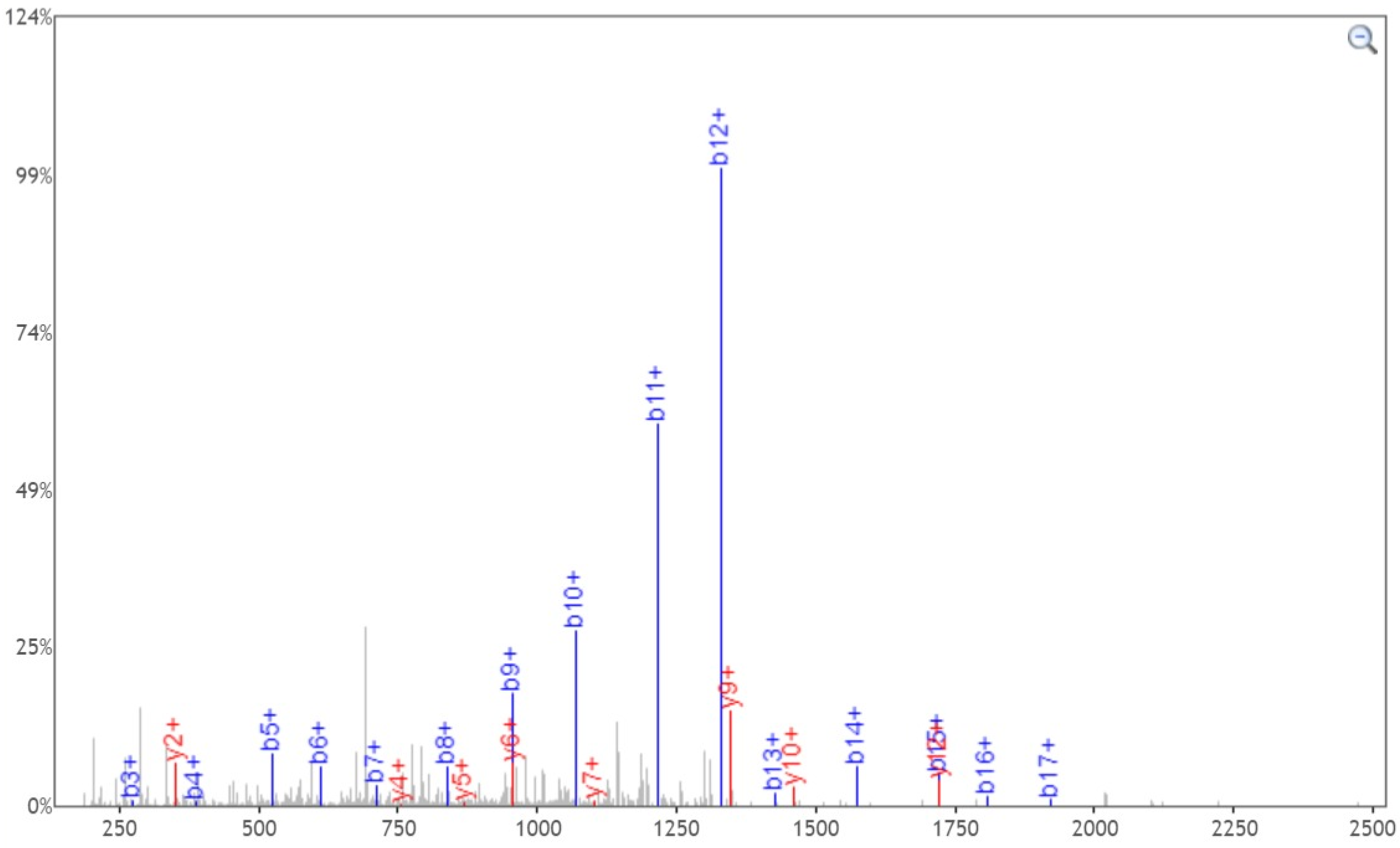

Click and drag in the plot to zoom X: Y: Zoom Out Print Enable tooltip

b+	#	Seq	#	y+
88.0393	1	S	21	
175.0713	2	S	20	2588.2708
274.1397	3	V	19	2501.2387
387.2238	4	L	18	2402.1703
524.2827	5	H	17	2289.0863
611.3148	6	S	16	2152.0274
712.3624	7	T	15	2064.9953
840.4210	8	Q	14	1963.9476
955.4480	9	D	13	1835.8891
1068.5320	10	L	12	1720.8621
1215.6004	11	F	11	1607.7781
1328.6845	12	L	10	1460.7096
1425.7373	13	P	9	1347.6256
1572.8057	14	F	8	1250.5728
1719.8741	15	F	7	1103.5044
1806.9061	16	S	6	956.4360
1920.9490	17	N	5	869.4040
2020.0175	18	V	4	755.3610
2324.1445	19	T	3	656.2926
2510.2238	20	W	2	352.1656
	21	F	1	166.0863

[Click] to move table

Static Modifications:
C: 57.02146

Variable Modifications:
T: 203.079373
