# Supplementary material for: Site-Specific O-Glycosylation Analysis of SARS-CoV-2 Spike Protein Produced in Insect and Human Cells
Source: Viruses. 2021 Mar 25;13(4):551. doi: 10.3390/v13040551 (PMC8064498; doi:10.3390/v13040551)
Supplement: Supplementary file 1 [file viruses-13-00551-s001.zip › Dataset S7. Spectra Q Exactive.zip!/spectrum list-Exp_1/T236-1-N.pdf]

Fragment Ion View

Project name: 20200330COV19
Sample name: 20200416_COV19-double_1
Created date: 2020-04-18 15:22:56.0
Search name:
Activation type: CID

Peptide sequence: R.EGVFVSN(2.988261)GTHWF.V
Scan number: 53387
Calculated M+H: 1382.6261
Measured M+H: 1382.631
Charge state: 2
File name: 20200416_COV19-double_1
search type: light

Ions:
a ☐1+ ☐2+ ☐3+
b ☑1+ ☐2+ ☐3+
c ☐1+ ☐2+ ☐3+
x ☐1+ ☐2+ ☐3+
y ☑1+ ☐2+ ☐3+
z ☐1+ ☐2+ ☐3+
[Deselect All]

Neutral Loss:
☐ H_2O (o)
☐ NH_3 (*)
☐ H_3PO_4 (^)
☐ Precursor (#)
☐ Precursor with reduced charges

Mass Type: ◉Mono ○Avg

Mass Tol: 20

○Dalton ◉PPM

Update

Peak Assignment:
◉Most Intense
○Nearest Match
☐Peak Detect
☐Precursor Removal

Peak Labels:
◉Ion ○m/z
○None

Width: 750

Height: 450

EGVFVSNGTHWF, MH+ 1382.6261, m/z 691.8167
File: 20200416_COV19-double_1, Scan: 53387, Precursor m/z: 691.81915, Charge: 2

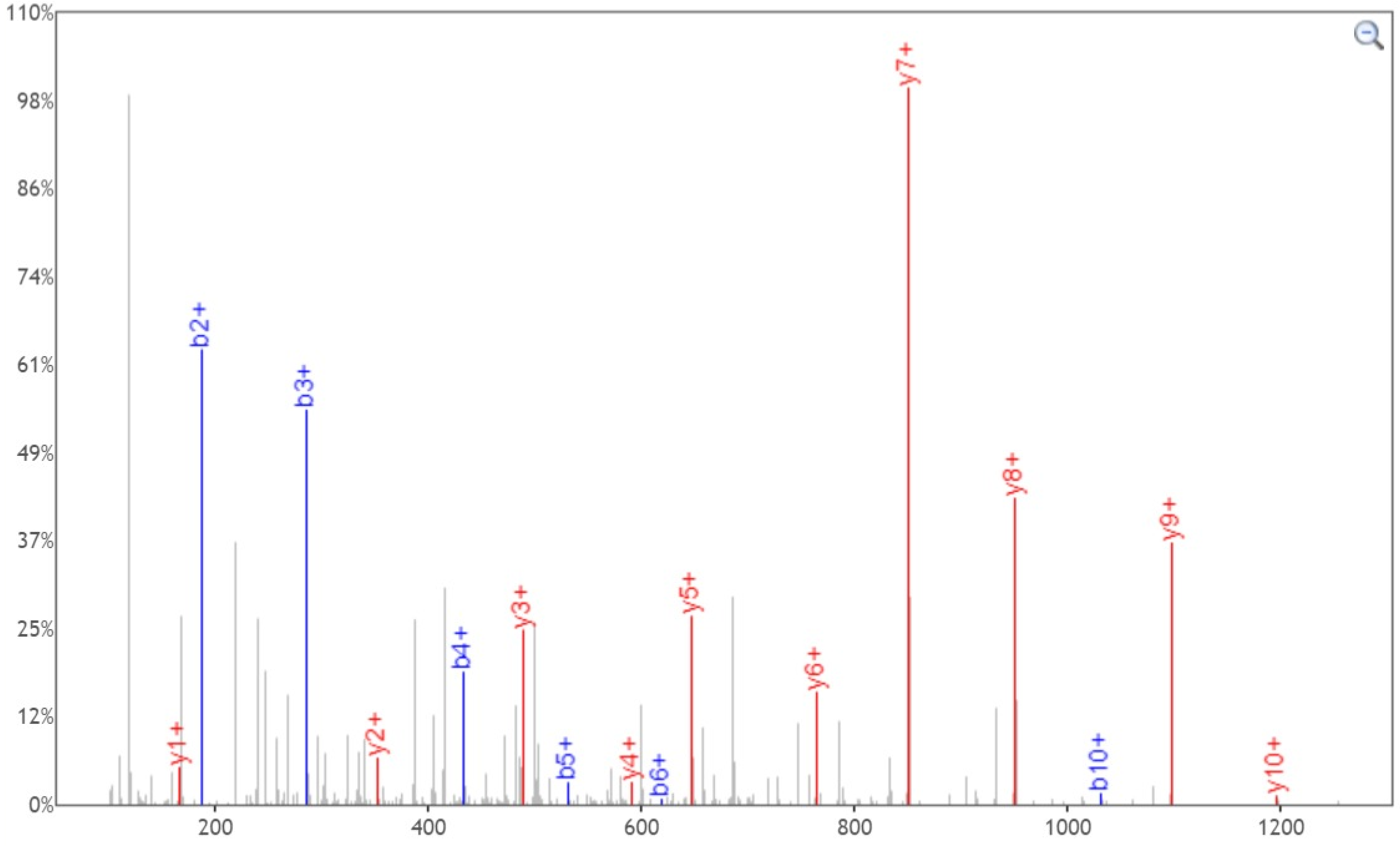

Click and drag in the plot to zoom X: ☑ Y: ☐ Zoom Out Print ☐Enable tooltip

b+	#	Seq	#	y+
130.0499	1	E	12	
187.0713	2	G	11	1253.5835
286.1397	3	V	10	1196.5621
433.2082	4	F	9	1097.4937
532.2766	5	V	8	950.4253
619.3086	6	S	7	851.3568
736.3398	7	N	6	764.3248
793.3613	8	G	5	647.2936
894.4089	9	T	4	590.2722
1031.4678	10	H	3	489.2245
1217.5472	11	W	2	352.1656
	12	F	1	166.0863

[Click] to move table

Static Modifications:
C: 57.02146

Variable Modifications:
N: 2.988261
