# Supplementary material for: Site-Specific O-Glycosylation Analysis of SARS-CoV-2 Spike Protein Produced in Insect and Human Cells
Source: Viruses. 2021 Mar 25;13(4):551. doi: 10.3390/v13040551 (PMC8064498; doi:10.3390/v13040551)
Supplement: Supplementary file 1 [file viruses-13-00551-s001.zip › Dataset S7. Spectra Q Exactive.zip!/spectrum list-Exp_1/T167-1-N.pdf]

Fragment Ion View

Project name: 20200330COV19
Sample name: 20200416_COV19-double_1
Created date: 2020-04-18 15:22:56.0
Search name:
Activation type: CID

Peptide sequence: R.VYSSANN(2.988261)CTFEYVSQPFLMDLEGK.Q
Scan number: 56061
Calculated M+H: 2802.2522
Measured M+H: 2802.2622
Charge state: 3
File name: 20200416_COV19-double_1
search type: light

Ions:
a ☐1⁺ ☐2⁺ ☐3⁺
b ☑1⁺ ☑2⁺ ☐3⁺
c ☐1⁺ ☐2⁺ ☐3⁺
x ☐1⁺ ☐2⁺ ☐3⁺
y ☑1⁺ ☑2⁺ ☐3⁺
z ☐1⁺ ☐2⁺ ☐3⁺
[Deselect All]

Neutral Loss:
☐ H_2O (o)
☐ NH_3 (*)
☐ H_3PO_4 (^)
☐ Precursor (#)
☐ Precursor with reduced charges

Mass Type:
◉ Mono ○ Avg

Mass Tol: 20
○ Dalton ◉ PPM

Update

Peak Assignment:
◉ Most Intense
○ Nearest Match
☐ Peak Detect
☐ Precursor Removal

Peak Labels:
◉ Ion ○ m/z
○ None

Width: 750

Height: 450

VYSSANNCTFEYVSQPFLMDLEGK, MH+ 2802.2523, m/z 934.7556
File: 20200416_COV19-double_1, Scan: 56061, Precursor m/z: 934.7589, Charge: 3

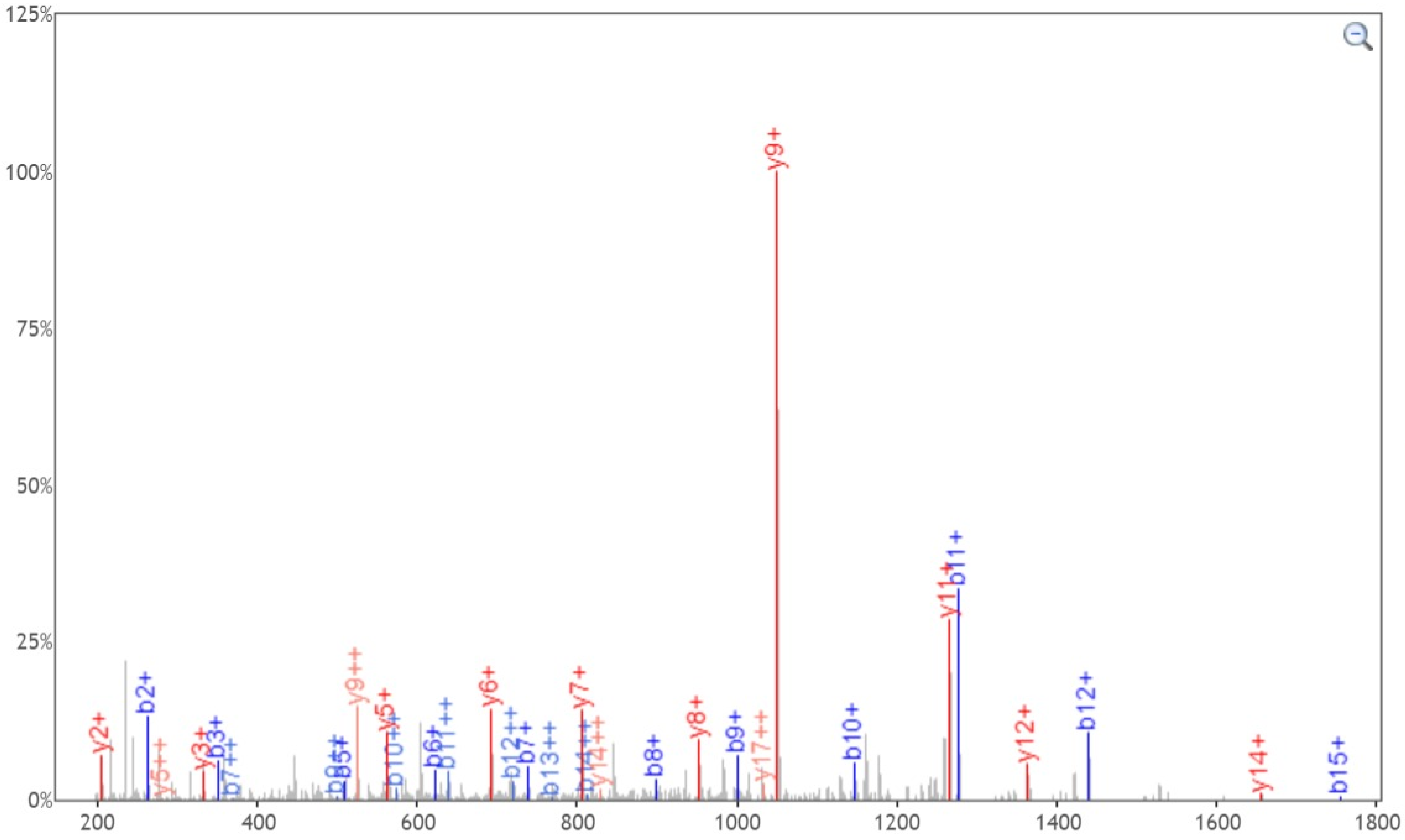

Click and drag in the plot to zoom X:☑ Y:☐ Zoom Out Print ☐Enable tooltip

b+	b2+	#	Seq	#	y+	y2+
100.0757	50.5415	1	V	24		
263.1390	132.0731	2	Y	23	2703.1839	1352.0956
350.1710	175.5892	3	S	22	2540.1206	1270.5639
437.2031	219.1052	4	S	21	2453.0885	1227.0479
508.2402	254.6237	5	A	20	2366.0565	1183.5319
622.2831	311.6452	6	N	19	2295.0194	1148.0133
739.3143	370.1608	7	N	18	2180.9765	1090.9919
899.3449	450.1761	8	C	17	2063.9453	1032.4763
1000.3926	500.7000	9	T	16	1903.9146	952.4610
1147.4610	574.2342	10	F	15	1802.8669	901.9371
1276.5036	638.7555	11	E	14	1655.7985	828.4029
1439.5670	720.2871	12	Y	13	1526.7559	763.8816
1538.6354	769.8213	13	V	12	1363.6926	682.3499
1625.6674	813.3373	14	S	11	1264.6242	632.8157
1753.7260	877.3666	15	Q	10	1177.5922	589.2997
1850.7787	925.8930	16	P	9	1049.5336	525.2704
1997.8472	999.4272	17	F	8	952.4808	476.7441
2110.9312	1055.9693	18	L	7	805.4124	403.2098
2241.9717	1121.4895	19	M	6	692.3284	346.6678
2356.9987	1179.0030	20	D	5	561.2879	281.1476
2470.0827	1235.5450	21	L	4	446.2609	223.6341
2599.1253	1300.0663	22	E	3	333.1769	167.0921
2656.1468	1328.5770	23	G	2	204.1343	102.5708
		24	K	1	147.1128	74.0600

[Click] to move table

Static Modifications:
C: 57.02146

Variable Modifications:
N: 2.988261
