# Supplementary material for: Site-Specific O-Glycosylation Analysis of SARS-CoV-2 Spike Protein Produced in Insect and Human Cells
Source: Viruses. 2021 Mar 25;13(4):551. doi: 10.3390/v13040551 (PMC8064498; doi:10.3390/v13040551)
Supplement: Supplementary file 1 [file viruses-13-00551-s001.zip › Dataset S7. Spectra Q Exactive.zip!/Dataset S7 legend.rtf]

Dataset S7. The zipped file contains spectra of O-glycosylated peptides included in occupancy quantification (Figure 2b and Dataset S6) of data derived from the Q Exactive HF-X experiments. Examples of non-O-glycosylated peptide spectra are also included. The naming of the files follows the following format O-glycosite position-number-Y/N, where the number indicates the order number in Dataset S6, and the Y/N informs, whether the peptide is O-glycosylated (Y - yes), or not (N - no).
